# AK4 promotes nasopharyngeal carcinoma metastasis and chemoresistance by activating NLRP3 inflammatory complex

**DOI:** 10.1038/s41419-025-07805-8

**Published:** 2025-07-01

**Authors:** Sai-Lan Liu, Li Yuan, Xue-Song Sun, Bei-Bei Xiao, Kai-Qi Lan, Zi-Jian Lu, Da-Feng Lin, Xiao-Yun Li, Jin-Jie Yan, Shu-Mei Yan, Qiu-Yan Chen, Lin-Quan Tang, Hai-Qiang Mai

**Affiliations:** 1 Sun Yat-sen University Cancer Center, State Key Laboratory of Oncology in South China, Collaborative Innovation Center for Cancer Medicine, Guangdong Key Laboratory of Nasopharyngeal Carcinoma Diagnosis and Therapy, Guangzhou, Guangdong Province People’s Republic of China; 2 Department of Nasopharyngeal Carcinoma, Sun Yat-sen University Cancer Center, Guangzhou, Guangdong Province People’s Republic of China; 3 Department of Critical Care Medicine, Sun Yat-sen University Cancer Center, Guangzhou, Guangdong Province People’s Republic of China; 4 Department of Pathology, Sun Yat-sen University Cancer Center, Guangzhou, Guangdong Province People’s Republic of China

**Keywords:** Head and neck cancer, Cancer genetics

## Abstract

Metastasis is the main cause of treatment failure in nasopharyngeal carcinoma (NPC). Our previous study developed a transcriptomics-based gene signature (AK4, CPAMD8, DDAH1, and CRTR1) to predict metastasis in NPC and identify candidates that could benefit from induction chemotherapy (IC). Of these, adenylate kinase 4 (AK4) is a potent oncogene involved in the malignant progression of a variety of tumors. This study investigated the expression and mechanism of action of AK4, a member of the AK family of enzymes, in NPC. Quantitative real-time PCR, western blotting, and immunohistochemistry revealed that AK4 was upregulated in NPC and correlated with metastasis and chemoresistance. Stable ectopic overexpression of AK4 in NPC cell lines conferred resistance to taxol-induced apoptosis, promoted the migration, invasion, and EMT phenotype, and induced IL-1β secretion by activating the NLRP3 signaling pathway; knockdown of AK4 had the opposite effects. Mechanistically, AK4 co-localized with NNT, upregulated NLRP3 and IL-1β, and consequently altered NPC cell metastasis and chemoresistance. AK4 may play a role in the development of NPC and represent a potential therapeutic target.

## Introduction

The incidence of nasopharyngeal carcinoma (NPC) is very high in southern China, and metastasis is the main cause of treatment failure in patients with NPC [1–3]. Several prospective randomized trials have demonstrated that induction chemotherapy (IC) followed by concurrent chemoradiotherapy (CCRT) reduces the risk of distant metastasis. However, our previous study showed that approximately 20% of patients are resistant to IC and at an increased risk of developing distant metastasis [4]. Therefore, distant metastasis and chemoresistance are significant obstacles to enhancing the survival rate of patients with NPC. Addressing these challenges is a pressing clinical issue requiring immediate attention. However, the molecular mechanisms underlying metastasis and chemoresistance of NPC remain unclear.

Emerging evidence has indicated that certain gene expression patterns can be used as molecular markers of metastasis and chemoresistance [5]. In our previous study, we performed transcriptome sequencing on NPC biopsy samples from 12 pairs of patients with different metastasis risks and identified 11 differentially expressed genes (DEGs). Four of these (AK4, CPAMD8, DDAH1, and CRTR1) were used to create a gene signature that effectively categorized patients into low-risk and high-risk metastasis groups and could be used to identify candidates who could benefit from IC + CCRT [6].

To further study the molecular mechanisms underlying metastasis and chemoresistance in NPC, we identified adenylate kinase 4 (AK4), a member of the AK family of enzymes, as the target gene. To date, nine known isoforms of AK, from AK1 to AK9 according to the order of discovery, have been identified [7, 8]. AK4 is localized in the mitochondrial matrix [9] and physically binds to the mitochondrial ADP/ATP translocase as a stress-responsive protein that protects cells from death [10]. Unlike other AK enzymes, AK4 is inactivated by enzymes in vitro but retains its nucleotide-binding ability [11]. AK4 is overexpressed in lung cancer [12], and high AK4 expression is associated with poor survival in many cancers [12–15]. Many studies have demonstrated that AK4 regulates the sensitivity of cancer cells to antitumor drugs [16–18] and radiation therapy [19, 20]. Additionally, AK4 expression is correlated with tumor invasion [21, 22]. Several studies have investigated the mechanisms underlying AK4 expression and tumor metastasis. Jan et al. [12]. demonstrated that AK4 promotes lung cancer metastasis by downregulating the expression of the transcription factor ATF3. Additionally, AK4 overexpression promotes lung cancer metastasis by enhancing hypoxia-inducible factor 1α (HIF-1α) stability and epithelial-to-mesenchymal transition (EMT) under hypoxia [23]. However, whether AK4 regulates metastasis and chemoresistance in NPC remains unclear.

In this study, we found that AK4 was highly expressed in NPC cells, and that high expression of AK4 significantly reduced the sensitivity of NPC cells to taxol, promoted the invasion and metastasis of NPC cells, and enhanced EMT in vitro and in vivo. Mechanistically, we found that IL-1β is a key downstream gene affected by AK4, and AK4 may promote IL-1β release by activating the NLRP3 inflammasome signaling pathway binding with nicotinamide nucleotide transhydrogenase (NNT), thereby promoting invasion, migration, and chemoresistance of tumor cells. Together, these findings demonstrate the important role of AK4 in mediating taxol resistance and metastasis in NPC.

## Materials and methods

### Patients and tumor tissue specimens

We obtained 108 paraffin-embedded specimens from the Sun Yat-sen University Cancer Center (SYSUCC), including 13 normal nasopharyngeal epithelial samples, 47 histologically and clinically diagnosed non-metastatic NPC samples, and 48 metastatic foci biopsy samples. In addition, we collected peripheral blood samples prior to any anticancer treatment from 262 patients with NPC, who were diagnosed at the SYSUCC between 2010 and 2017. The eighth edition of the American Joint Committee on Cancer Staging Manual was used for restaging of all patients.

### RNA extraction, cDNA library preparation, RNA-Seq, and RT-PCR

RNA extraction, cDNA library preparation, and RNA-Seq were performed in accordance with our previous study [6]. RT-PCR was conducted as previously described [24] using primers designed using Primer Express Software (version 2.0; Applied Biosystems). Target gene expression data were normalized to that of GAPDH, and all experiments were performed in triplicate. The primer sequences used are listed in Supplementary Table 1.

### Microarray data process and gene set enrichment analysis (GSEA)

Microarray data processing and visualization of microarray datasets (GEO accession numbers: GSE12452 and GSE53819) from the NPC and control samples were retrieved from the GEO database (http://www.ncbi.nlm.nih.gov/geo/). Subsequently, we performed an integrative analysis of The Cancer Genome Atlas head and neck tumor and normal tissue microarray data (https://cancergenome.nih.gov/). Gene expression profiles of 24 cases of NPC were used to conduct GSEA (http://software.broadinstitute.org/gsea/msigdb) [25] to identify gene signatures between groups with high and low AK4 expression levels.

### Cell lines, cell culture, plasmids, transfection, and RNA interference

Human NPC cells CNE1, CNE2, HK1, HONE1, HNE1, SUNE1, SUNE2, and CNE2 subclones S18 and S26, as well as SUNE1 subclones 5-8 F and 6-10B, were maintained in RPMI-1640 (Invitrogen, Grand Island, NY, USA) and 5% fetal bovine serum (FBS; HyClone, Logan, UT, USA); NP69 immortalized nasopharyngeal epithelial cells were cultured in keratinocyte/serum-free medium (Invitrogen), and 293FT cells were maintained in DMEM (Gibco, Grand Island, NY, USA) supplemented with 10% FBS at 37 °C with 5% CO_2_. The cells were treated with interleukin (IL)-1β or interleukin 1 receptor antagonist (IL-1Ra) or transfected with a recombinant lentivirus carrying a human AK4 overexpression plasmid, shRNA, or corresponding empty vectors (Genecopoeia, Guangzhou, China). Effective siRNA oligonucleotides targeting NLRP3 and NNT were obtained from RiboBio (Guangzhou, China). All primers and oligonucleotides used for plasmid construction are listed in Supplementary Table 2 (Primers and Oligonucleotides). 293FT cells were co-transfected with lentivirus packaging expression plasmids to generate stably transfected cell lines. The lentiviral particles were subsequently harvested, and they were infected into NPC cells after 48 h. Stable clones were then selected using 0.5–1 μg/mL puromycin (Sigma-Aldrich), and RT-PCR or western blotting assays were used to validate the infection efficiency.

### Western blotting and immunohistochemistry (IHC)

Western blotting [24] was performed using anti-AK4 rabbit polyclonal antibody (1:1000; Proteintech); antibodies against E-cadherin, vimentin, IL-1β, ASC, NNT (1:1000, Abcam), NLRP3 (1:1000, Adipogen), procaspase-1, caspase-1, and pro-IL-1β (1:1000, Proteintech); and anti-α-tubulin monoclonal antibody (1:2000; Proteintech). For correlation analysis, NPC tissue sections were subjected to IHC staining [24] using an anti-AK4 polyclonal antibody (1:100; Proteintech), IL-1β (1:200; Proteintech) and NNT (1:100; Abcam) at 4 °C overnight; normal goat serum was used as the negative control. The degree of immunostaining was scored independently by two observers in a blinded manner. For histological evaluation, mouse lung metastatic nodules were resected, fixed in 4% paraformaldehyde, and routinely processed.

### Wound healing, transwell migration and invasion assay, and ELISA

Transfected cells (1 × 10^6^) were seeded, allowed to reach 70–80% confluence, and subsequently starved for 24 h. The cell monolayers were then wounded with a sterile plastic tip and cultured in serum-free medium. Cell migration was monitored every 12 h under a microscope (Nikon).

Stable cells (6 × 10^4^) were seeded on top of a thick layer with or without Matrigel in transwell inserts (BD Biosciences) and cultured for another 24 h. Invasive cells adhering to the lower surface of the filter were washed with phosphate-buffered saline (PBS), fixed in 4% paraformaldehyde, and stained with 0.05% crystal violet. Invasive cells were counted under a light microscope (Zeiss).

ELISA was performed using the IL-1β ELISA kit (ab214025; Abcam) according to the manufacturer’s instructions.

### Drug treatment and CCK-8 assay

Cells were seeded in triplicate into 96-well plates (1 × 10^4^ cells/well; 100 µl medium).

Cells were cultured for 96 h and then incubated with 0, 2, 4, 6, 10, and 16 nM taxol (Hospira Australia Pty Ltd., Haikou, China) for 24 h. CCK-8 solution (20 µl; 5 mg/ml) was added 4 h before the end point; 200 µl of DMSO was added to each well, and absorbance was measured at 450 nm. All experiments were performed in triplicate.

### Annexin-V and TUNEL assay

Cells (1 × 10^5^) were seeded in triplicate into 6-well plates and harvested 24 h later.

Cells were treated with taxol for 72 h, stained using the Annexin V-FITC apoptosis detection kit (Beyotime), and immediately analyzed using the FSCAN flow cytometer (BD Biosciences, USA). Slices from xenograft tumors were stained using the RiboAPO One-Step TUNEL Apoptosis Kit to detect apoptotic cells.

### ROS and measurement of NADPH

Lipid reactive oxygen species (ROS) production was detected by flow cytometry using C11-BODIPY dye (#D-3861; Life Technologies, Grand Island, NY, USA) according to the manufacturer’s instructions. Briefly, cells were seeded into 12-well plates and cultured in a 37 °C incubator with 5% CO_2_. The cells were then treated with different cytotoxic reagents for the indicated times, C11-BODIPY was added to the cell supernatants, and the cells were cultured for more than 30 min before ROS detection. ROS can oxidize the polyunsaturated butadienyl portion of C11-BODIPY, and then the fluorescence emission peak of the dye shifted from ~590 nm to ~510 nm. The NADPH/NADP+ ratio was determined using an NADP + /NADPH Assay Kit with WST-8 (S0179, Beyotime, China) according to the manufacturer’s instructions as described previously [26].

### Mass spectrometry and co-immunoprecipitation (co-IP) assay

For immunoprecipitation (IP) assays, 6-10B cells were lysed with IP lysis buffer. Primary anti-AK4 (2 μg; Proteintech) or anti-IgG (negative control, 3 μg; Proteintech) antibodies were incubated with the lysates at 4 °C overnight. Protein A/G Sepharose beads (Thermo Fisher Scientific) were used to recover the immune complexes, which were washed and collected after isolation. Mass spectrometry analysis was performed by Huijun Biotechnology (China). For the co-IP assay, western blotting was performed to determine the protein levels.

### Immunofluorescence staining

For the immunofluorescence staining assay, the cells were cultured on coverslips (Thermo Fisher Scientific). After 24 h, the cells were fixed in 2% paraformaldehyde, permeabilized with 0.5% Triton X-100 in PBS, and incubated with primary anti-AK4 (1:100; Proteintech) and anti-NNT (1:200; Abcam) antibodies at 4 °C overnight. The coverslips were incubated with Alexa Fluor 488 and 594 goat IgG secondary antibodies (1:1,000; Abcam) and counterstained with 4′, 6-diamidino-2-phenylindole. Images were captured using a confocal laser scanning microscope (Olympus FV1000, Tokyo, Japan).

### In vivo lung metastasis model and xenograft model

Female BALB/c nude mice (4 weeks old) were purchased from the Guangdong Medical Laboratory Animal Center (Guangzhou, China). For the lung metastasis model, SUNE2-AK4/SUNE2-Vector cells or 5–8F-Ri-Vector/5-8F-AK4-Ri1 cells were resuspended in PBS, and 1 × 10^6^ cells were injected into the tail vein of the mice (*n* = 6 per group). Eight weeks later, the mice were sacrificed, and lung metastatic colonies were quantified. For IL-1Ra treatment of the lung metastasis model, SUNE2 cells with or without AK4 overexpression (1 × 10^6^ cells in 100 μl PBS) were injected into the tail vein of mice. Two weeks later, 100 μl of saline or IL-1Ra at a dose of 1 mg/kg/day was delivered intraperitoneally for 21 days. Three weeks later, the mice were sacrificed for further analysis.

For the xenograft model, female BALB/c nude mice were randomly divided into two groups (*n* = 12) and subcutaneously inoculated with SUNE2-AK4/SUNE2-Vector cells or 5-8F-Ri-Vector/5-8F-AK4-Ri1 cells (1 × 10^6^; 100 μl sterile PBS). Tumor volumes were measured every three days ([length × width^2^]/2). When tumors became palpable (approximately 100 mm^3^), the mice were intraperitoneally injected with 100 μl dimethyl sulfoxide or 5 mg/kg taxol (*n* = 6 per group) every two days. On day 31, the primary tumors were carefully removed, imaged, sliced, and analyzed using IHC and TUNEL assays.

All animal experiments were performed according to the protocols of the Institutional Animal Care and Use Committee of Sun Yat-sen University Cancer Center. All experimental methods complied with the guidelines of the Declaration of Helsinki.

### Statistical analysis

All data are presented as the mean ± SEM from at least three independent experiments. A two-tailed Student’s *t*-test was used for comparisons between groups. Comparisons among categorical variables were performed with χ^2^ and Fisher’s exact tests.

Kaplan–Meier analysis and the log-rank test were used to compare the survival rates. Multivariate analysis was performed using the Cox proportional hazards model. All statistical analyses were performed using R (version 3.6.0), SPSS (version 22.0; IBM Corp., Armonk, NY, USA), and GraphPad Prism 5. All statistical tests were two-sided, and statistical significance was set at *p* < 0.05.

## Results

### AK4 is upregulated in NPC and correlated with metastasis and chemoresistance

**Fig. 1:**
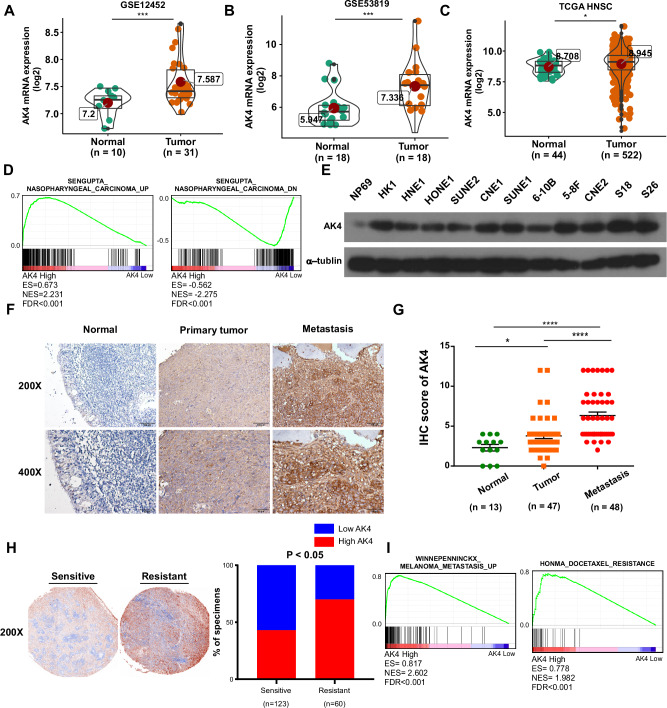
AK4 is upregulated in nasopharyngeal carcinoma (NPC) and correlated with metastasis and chemoresistance. AK4 expression in NPC tumor and normal tissue samples in array express microarray data (GEO accession number: GSE12452 and GSE53819; http://www.ncbi.nlm.nih.gov/geo/) (**A**, **B**), The Cancer Genome Atlas (head and neck) tumor, and normal tissue microarray data (https://cancergenome.nih.gov/) (**C**). GSEA plot showing that AK4 expression is positively correlated with NPC-activated gene signatures (SENGUPTA_NASOPHARYNGEAL_CARCINOMA_UP) and inversely correlated with NPC-suppressed gene signatures (SENGUPTA_NASOPHARYNGEAL_CARCINOMA_DN) in our previous nasopharyngeal carcinoma gene expression profiles (*n* = 24) (**D**). GSEA was performed using GSEA 4.0.3 (http://www.broadinstitute.org/gsea/). Western blotting of AK4 protein expression in NP69 immortalized nasopharyngeal epithelial cells and eleven cultured NPC cell lines (**E**). Representative images of immunohistochemical staining for AK4 in normal nasopharyngeal epithelial biopsies, low and high AK4 expression in NPC tissues, and metastatic tissues of patients with NPC (**F**, **G**). Representative images of immunohistochemical staining of AK4 in chemosensitive and chemoresistant NPC tissues from our previous study (**H**). GSEA plot showing that AK4 expression is positively correlated with metastasis-activated gene signatures (WINNEPENNINCKX_MELANOMA_METASTASIS_UP) and inversely correlated with metastasis-suppressed gene signatures (WINNEPENNINCKX_MELANOMA_METASTASIS_DN) in our previous NPC gene expression profiles (*n* = 24) (**I**).

Tumor and normal tissue microarray data (NPC, GSE12452, GSE53819; head and neck tumor, the Cancer Genome Atlas) revealed that AK4 was the only gene that was upregulated in both NPC and head and neck tumor samples compared to normal samples among the gene signatures (AK4, CPAMD8, DDAH1, and CRTR1) (Fig. 1A, B, C; Supplementary Fig. 1A, B, C). Furthermore, high expression of AK4 was correlated with worse prognosis in patients with head and neck cancer patients compared with low expression of AK4 (Supplementary Fig. 1D). Additionally, our re-analysis of previously published data^6^ showed that patients with nasopharyngeal carcinoma with high AK4 expression have poor prognosis (Supplementary Fig. 1E). No correlation was found between AK4 expression and stage, grade, or TNM classification (Supplementary Table 2). GSEA [25] of our published gene expression profiles of 12 pairs of patients with different metastasis risks demonstrated that AK4 expression correlated positively with NPC-up gene signatures and inversely with NPC-down gene signatures (Fig. 1D). Western blotting revealed that AK4 was upregulated in all 11 NPC cell lines compared to immortalized nasopharyngeal epithelial NP69 cells (Fig. 1E). IHC results showed that AK4 expression was higher in metastatic and primary nasopharyngeal tissues than in normal nasopharyngeal tissues. Interestingly, we found that the expression of AK4 was still higher in metastatic lesions than in primary lesions (Fig. 1F, G). In addition, AK4 was found to be significantly upregulated in chemoresistant patients compared with chemosensitive patients by reanalysis of our previous data [6] (Fig. 1H), suggesting a correlation between AK4 upregulation and the clinical response to IC in NPC. Increasing evidence has demonstrated that taxol-based IC can bring survival benefits to nasopharyngeal carcinoma patients. Furthermore, GSEA of our previous gene expression profiles revealed that AK4 expression positively correlated with metastasis and docetaxel resistance gene signatures (Fig. 1I). Taken together, these results suggest that AK4 expression is correlated with metastasis and chemoresistance in NPC.

### AK4 confers resistance to taxol-induced apoptosis in vitro

**Fig. 2:**
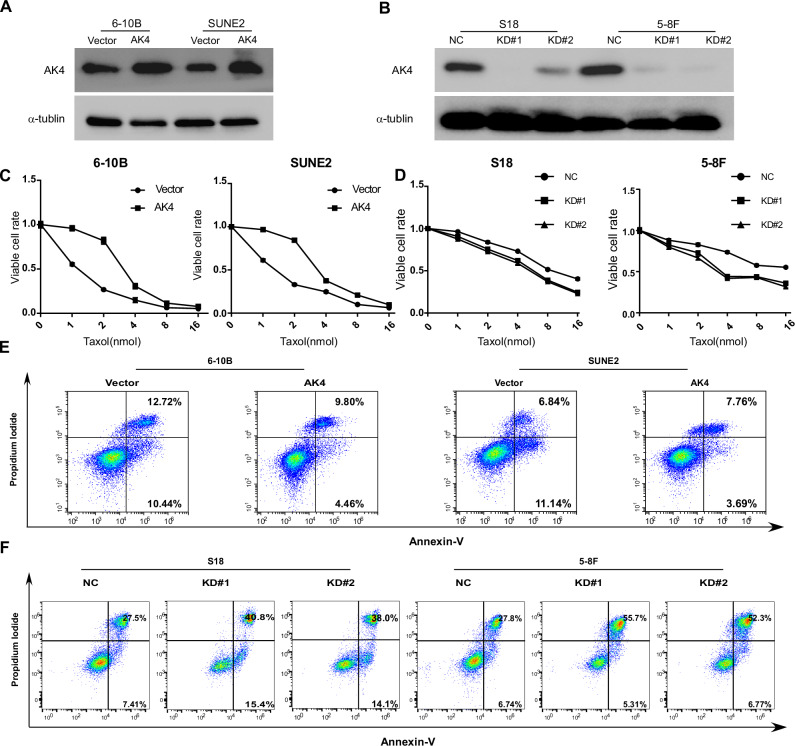
AK4 confers resistance to taxol-induced apoptosis in vitro. **A** Western blot analysis of ectopic AK4 overexpression in NPC cell lines (**A**). Western blot analysis of AK4 knockdown using specific shRNAs (**B**). CCK-8 assay for the viability of AK4-overexpressing (**C**) and AK4-knockdown (**D**) cells after 72 h of treatment with the indicated concentrations of taxol. Flow cytometry analysis of the percentage of apoptotic cells (Annexin V^+^/PI cells and Annexin V^+^/PI^+^ cells) in the indicated cell lines after 72 h of treatment with taxol (**E**, **F**).

The above results suggest AK4 as a key molecule in NPC metastasis and chemoresistance; however, whether AK4 affects NPC metastasis and chemoresistance remains unknown. We selected the 6-10B and SUNE2 cell lines for AK4 overexpression due to their relatively low endogenous expression levels of AK4 (Fig. 2A). In contrast, we chose the 5–8 F and S18 cell lines for AK4 knockdown, as they exhibit high endogenous expression of AK4 (Fig. 2B). Compared to control cells, AK4 overexpression conferred taxol resistance (Fig. 2C) and attenuated taxol-induced apoptosis (Fig. 2E). Conversely, AK4 knockdown increased taxol sensitivity (Fig. 2D) and the number of viable and non-viable apoptotic cells (Fig. 2F).

### AK4 promotes the migration, invasion, and EMT of NPC cells in vitro

**Fig. 3:**
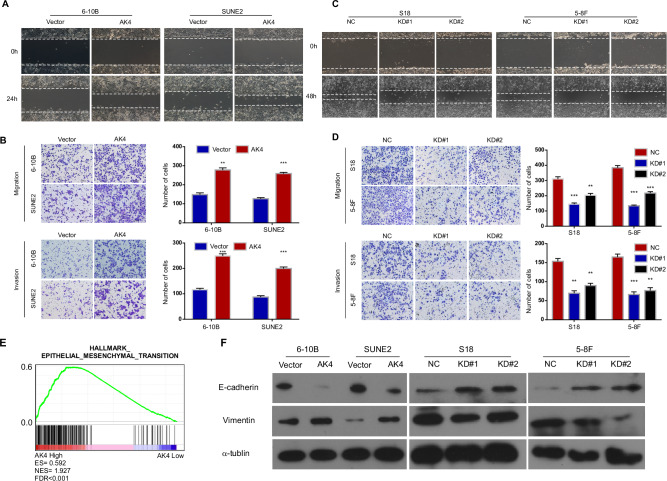
AK4 promotes the migration, invasion, and EMT of NPC cells in vitro. AK4 overexpression promotes 6-10B and SUNE2 cell migration and invasion as determined using wound healing and transwell invasion assays (**A**, **B**). AK4 knockdown inhibits S18 and 5–8 F cell migration and invasion as determined using wound healing and transwell invasion assays (**C**, **D**). Gene set enrichment analysis (GSEA) plot showing AK4 expression is positively correlated with EMT-activated gene signatures (HALLMARK_EPITHELIAL_MESENCHYMAL_TRANSITION) in published gene expression profiles of 31 cases of NPC (NCBI/GEO/GSE12452) (**E**). Western blot analysis for E-cadherin and vimentin in 6-10B-vector, 6-10B-AK4, SUNE2-vector, SUNE2-AK4, S18-vector, S18-AK4-KD#1 and S18-AK4-KD#2, and 5-8F-AK4-KD#1 and 5–8 F -AK4-KD#2 cells; α-tubulin was used as the loading control (**F**). Data are represented as the mean ± SEM. * *p* < 0.05, *** *p* < 0.001, **** *p* < 0.0001; Student’s *t* test.

As shown in Fig. 3A, AK4 overexpression significantly increased cell mobility compared to vector control cells. Additionally, compared to the control cells, AK4 overexpression significantly increased the number of migratory and invasive cells (Fig. 3B). Conversely, AK4 knockdown reduced the invasiveness and migration of NPC cells (Fig. 3C, D). In addition, GSEA of our previous gene expression profiles demonstrated that AK4 expression positively correlated with EMT gene signatures (Fig. 3E). Western blotting revealed that AK4 overexpression significantly downregulated the epithelial marker E-cadherin and upregulated the mesenchymal marker vimentin (Fig. 3F). Conversely, AK4 knockdown significantly downregulated E-cadherin expression and upregulated vimentin expression (Fig. 3F). Collectively, these data indicate that AK4 promotes EMT of NPC cells in vitro.

### AK4 promotes chemoresistance and metastasis in vivo

**Fig. 4:**
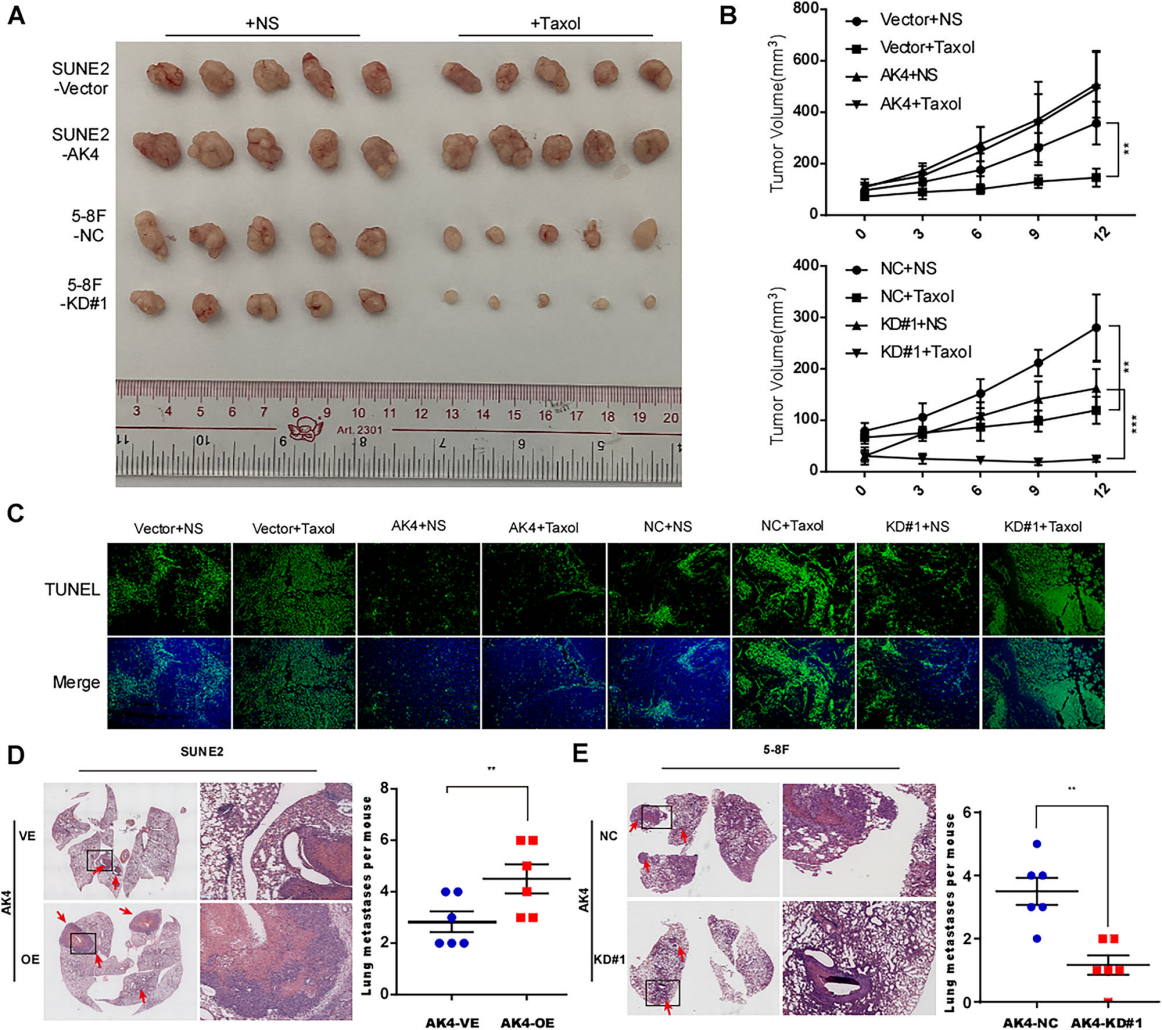
AK4 promotes chemoresistance and metastasis in vivo. Tumors formed by AK4-transduced SUNE2 cells were larger than the vector control tumors. Conversely, tumors formed by AK4-silenced 5–8 F cells were smaller than those formed by the vector cells (**A**). Tumor volume growth curve (**B**). Representative images of immunofluorescence staining of TUNEL-stained cells in the indicated tumors (**C**). SUNE2-vector, SUNE2-AK4, 5–8F-NC, and 5–8 F shAK4#1 cells (1 × 10^6^ cells in 100 µl PBS) were injected into the tail vein of mice. Mice in the AK4 group displayed a significantly higher number of metastatic lung nodules than mice in the control group and vice versa. Representative images and quantification of metastatic nodules in the lungs of mice (**D**, **E**). Data are represented as the mean ± SEM. ** *p* < 0.01, *** *p* < 0.001; Student’s *t* test.

To examine the effects of AK4 on chemoresistance in vivo, nude mice were subcutaneously injected with SUNE2 and 5–8 F cells. When the tumors reached a volume of approximately 100 mm^3^, the animals were randomly assigned to receive intraperitoneal injections of DMSO (control) or taxol. Interestingly, the volume of tumors formed by SUNE2-AK4 cells was not significantly affected by taxol treatment (Fig. 4A). However, taxol inhibited the growth of tumors formed by vector control cells and AK4 knockdown cells (Fig. 4B). Consistent with these results, tumors in the SUNE2-AK4 group contained significantly fewer apoptotic cells than those in the other groups (Fig. 4C), strongly suggesting that AK4 confers resistance to taxol in NPC. To evaluate the effect of AK4 on NPC metastasis in vivo, we used a lung metastasis colonization model. Hematoxylin and eosin staining confirmed that mice with AK4 knockdown exhibited remarkably fewer and smaller metastatic tumors in the lungs (Fig. 4D, E, *p* < 0.01).

### Transcriptome analysis reveals that IL-1β is a downstream target affected by AK4

To elucidate the molecular mechanisms by which AK4 contributes to the invasive phenotype of NPC cells, we performed transcriptome analysis to compare the gene expression of 5–8 F NC versus 5–8 F shAK4 cells and S18 NC versus S18 shAK4 cells.

**Fig. 5:**
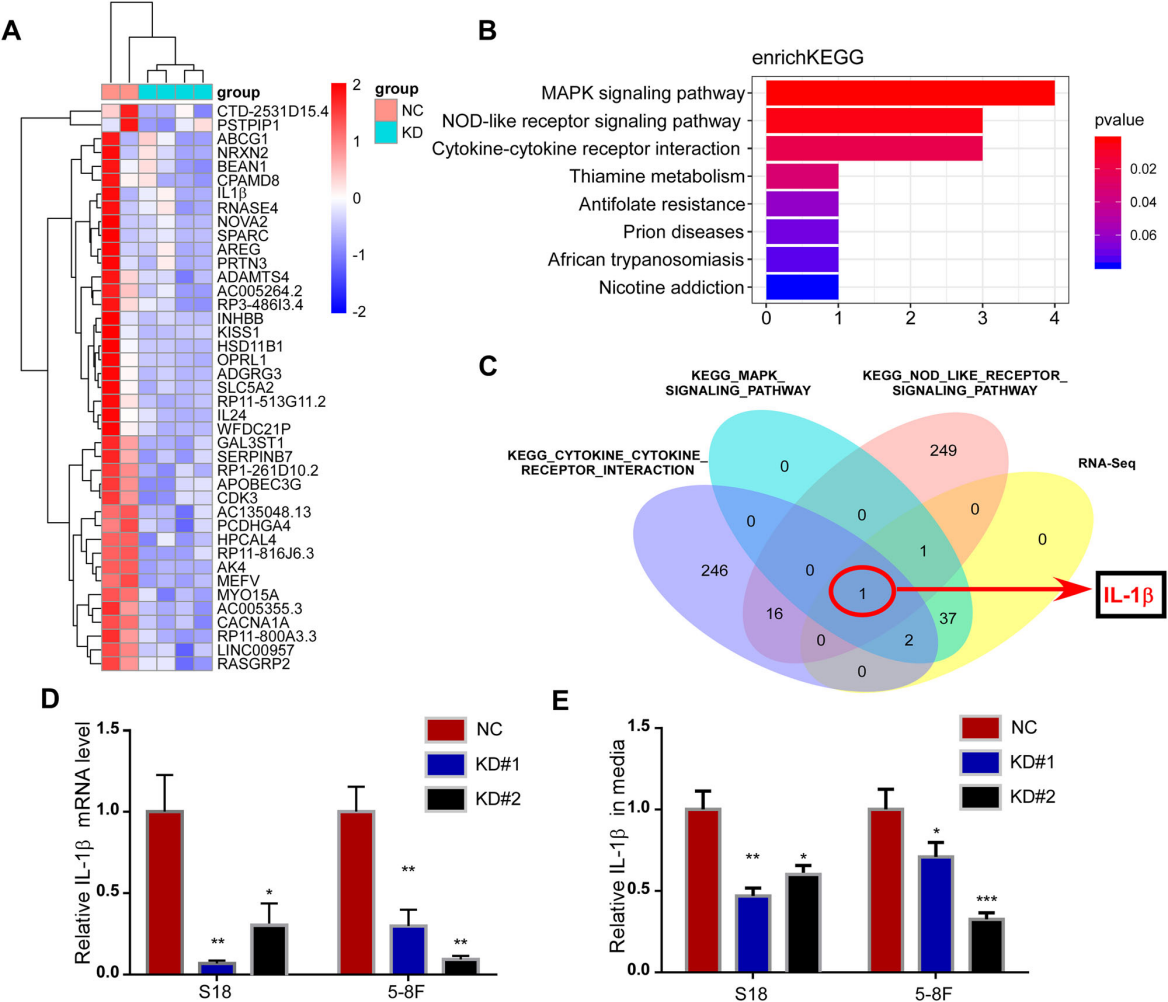
Transcriptome analysis reveals that IL-1β is a downstream target affected by AK4. Heat-map analysis of 41 downregulated genes screened out between S18 NC versus S18 shAK4#1, S18 shAK4#2 cells and 5–8 F NC versus 5–8 F shAK4#1, 5–8 F shAK4#2 cells (**A**). Kyoto Encyclopedia of Genes and Genomes pathway determined the MAPK signaling pathway, NOD-like receptor signaling pathway, and cytokine receptor interaction gene sets, which were significantly enriched in DEGs regulated by AK4 (**B**). Venn diagram analysis shows that IL-1β is a key molecule in the above three signaling pathways (**C**). The mRNA expression level of IL-1β in AK4 knockdown cells was significantly decreased (**D**). The expression levels of IL-1β in the conditioned medium of AK4 knockdown cells was significantly decreased (**E**). Data are represented as the mean ± SEM. * *p* < 0.05, ** *p* < 0.01, *** *p* < 0.001; Student’s *t* test.

According to the conditions (log2Fc = 2, *p* < 0.05), 41 downregulated genes were identified between the four treatment groups and the control group (Fig. 5A).

Next, we conducted gene annotation enrichment analysis using the Kyoto Encyclopedia of Genes and Genomes database to determine whether particular gene sets are significantly enriched in DEGs regulated by AK4.

As shown in Fig. 5B, DEGs were mainly concentrated in signaling pathways such as the MAPK signaling pathway, NOD-like receptor signaling pathway, and cytokine receptor interaction. Venn diagram analysis further identified that IL-1β was a downstream target affected by AK4 and a key molecule in the above three signaling pathways (Fig. 5C). Furthermore, the mRNA expression level of IL-1β in AK4 knockdown cells was significantly decreased (Fig. 5D). Additionally, ELISA results also showed that the expression levels of IL-1β in the conditioned medium of NPC cells were influenced by AK4 (Fig. 5E).

### AK4 promotes NPC cell metastasis and chemoresistance by regulating IL-1β secretion

**Fig. 6:**
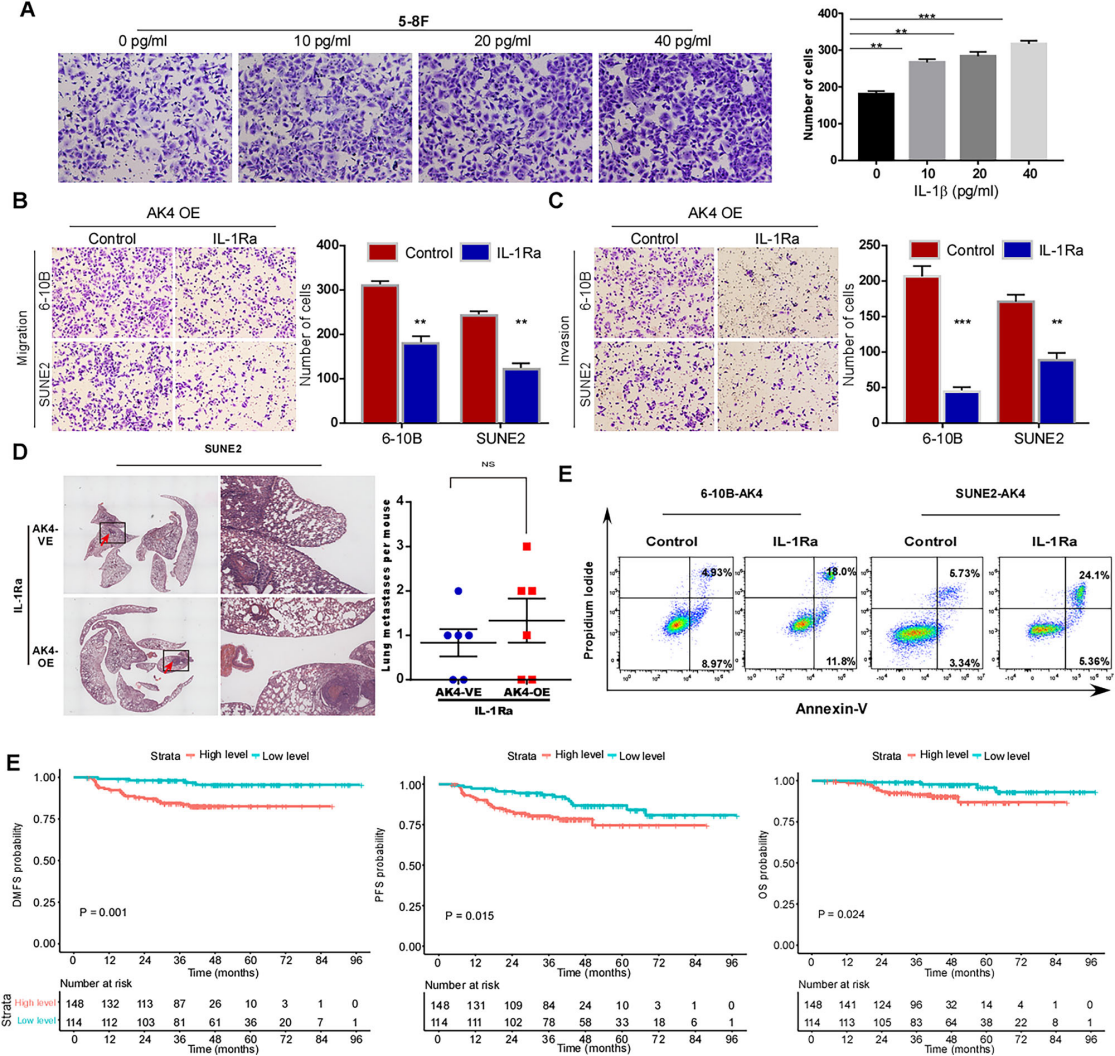
AK4 promotes NPC cell metastasis by regulating IL-1β secretion. The promoting effect of IL-1β on NPC migration increased with the increase in concentration (**A**). Inhibition of IL-1β signaling using IL-1Ra partially abrogated AK4-promoted migration, invasion, and resistance to taxol-induced apoptosis in vitro (**B**–**E**). IL-1Ra inhibited lung metastasis in the vector control cells and AK4 overexpressing cells (**D**). Survival analysis showed that the 5-year DMFS, PFS, and OS rates of patients with NPC and higher serum IL-1β levels were significantly lower than those of patients with NPC and lower IL-1β concentration (**E**). Data are represented as the mean ± SEM. * *p* < 0.05; ** *p* < 0.01; *** *p* < 0.001; Student’s *t* test.

IL-1β is a member of the IL-1 family, whose main function is to participate in inflammatory responses as an endogenic heat source [27]. Recent studies have shown that IL-1β plays an important role in tumor metastasis [28–34]. The transwell assay showed that the number of sublevel cells in the control group was significantly lower than that in the treatment group (10 pg/ml, 20 pg/ml, and 40 pg/ml) after 24 h of IL-1β treatment, and the difference between the treatment and control groups was statistically significant (p < 0.05). Additionally, the effect of IL-1β on NPC migration increased with the increase in concentration (Fig. 6A). In summary, the inflammatory factor IL-1β can promote the migration of NPC 5-8 F cells in a concentration-dependent manner. However, whether AK4 promotes metastasis through IL-1β in NPC cells has not been studied to date; therefore, we conducted the following studies. Inhibition of IL-1β signaling with IL-1Ra partially abrogated AK4-promoted migration, invasion, and chemoresistance (Fig. 6B–E), indicating that IL-1β signaling is a functional mediator of the ability of AK4 to promote migration, invasion, and resistance to taxol-induced apoptosis in vitro. Additionally, as shown in Fig. 6D, IL-1Ra inhibited lung metastasis in the vector control cells and AK4 overexpressing cells (p > 0.05).

Further, we used ELISA to analyze the expression of IL-1β protein in the serum samples of 262 patients with NPC. The ROC value of IL-1β concentration (31.3 pg/ml) was taken as the cut-off value, and it was divided into high and low concentration groups. The results showed that the high concentration of IL-1β in serum was positively correlated to the T stage of NPC (*p* = 0.035) (Supplementary Table 3). Multivariate analysis confirmed AK4 expression was an independent prognostic factor for DMFS (*P* = 0.001, Supplementary Table 4). Additionally, survival analysis showed that the 5-year distant metastatic rate of patients with NPC and higher serum IL-1β levels was significantly lower than that of patients with NPC and lower IL-1β concentration, indicating that patients with NPC and a higher serum IL-1β concentration were prone to metastasis (Fig. 6E).

In addition, microarray analysis (GSE13597, GSE53819, and TCGA) revealed that AK4 expression was positively correlated to IL-1β expression in NPC and head and neck tumor samples (Supplementary Fig. 2A, B, C). Additionally, IHC was performed to detect AK4 and IL-1β expression in the same cohort of NPC samples (*n* = 47). The results revealed a significant positive correlation between IL-1β and AK4 expression (r = 0.331, *p* = 0.023, Supplementary Fig. 2D, E). Western blotting further revealed the positive association between AK4 and IL-1β in NPC samples (r = 0.803, *p* = 0.001, Supplementary Fig. 2F, G). These results strongly suggest that AK4 promotes metastasis and chemoresistance in NPC via IL-1β, and IL-1β might be a potential therapeutic target for NPC.

### AK4 induces IL-1β secretion by activating the NLRP3 signaling pathway

The production of active, mature IL-1β is mainly dependent on the cleavage of inactive pro-IL-1β precursor by caspase-1, which is activated in a large multiprotein complex called the inflammasome. The NLRP3 inflammasome, comprising the NOD-like receptor protein NLRP3, the adaptor protein ASC, and pro-caspase-1, is the most important inflammasome involved in IL-1β processing [35]. Furthermore, GSEA revealed that DEGs were mainly concentrated in the NOD-like receptor signaling pathway and cytokine receptor interaction, which indicated that AK4 might induce IL-1β secretion through the NLRP3 signaling pathway.

**Fig. 7:**
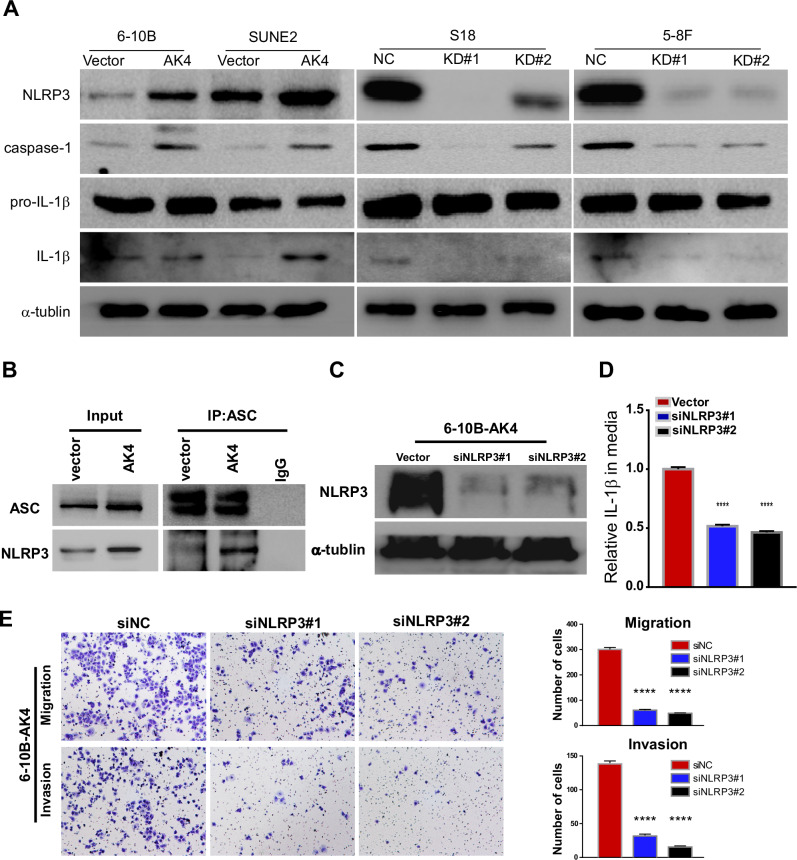
AK4 induces IL-1β secretion by activating the NLRP3 signaling pathway. Western blot revealed that AK4 overexpression upregulated the expression of NLRP3, caspase-1, and IL-1β, and AK4 knockdown in cells reduced the expression of NLRP3, caspase-1, and IL-1β (**A**). AK4 overexpression enhanced the interaction between NLRP3 and ASC (**B**). Western blot showed that NLRP3-targeting siRNAs were transfected into 6-10B cells stably overexpressing AK4 (**C**). NLRP3 knockdown abrogated AK4-mediated IL-1β release and promotion of migration and invasion (**D**, **E**). Data are represented as the mean ± SEM. * *p* < 0.05, ** *p* < 0.01, *** *p* < 0.001, **** *p* < 0.0001; Student’s *t* test.

Our western blotting results revealed that AK4 overexpression upregulated the expression of NLRP3, caspase-1, and IL-1β, whereas AK4 knockdown in cells reduced the expression levels of NLRP3, caspase-1, and IL-1β (Fig. 7A). In addition, AK4 overexpression increased the binding affinity of NLRP3 to ASC, indicating that AK4 promoted the NLRP3/ASC interaction (Fig. 7B). Next, we determined whether NLRP3 inflammasome activation is required for AK4-mediated IL-1β release and pro-metastatic effects on NPC cells. NLRP3-targeting siRNAs were transfected into 6-10B cells stably overexpressing AK4 (Fig. 7C). The results showed that NLRP3 knockdown significantly abrogated AK4-mediated IL-1β release and promoted its migration and invasion (Fig. 7D, E). The results also demonstrated that AK4 activated the NLRP3 signaling pathway, which further led to IL-1β release and promotion of migration and invasion.

### AK4 might regulate NLRP3 signaling by binding to NNT

**Fig. 8:**
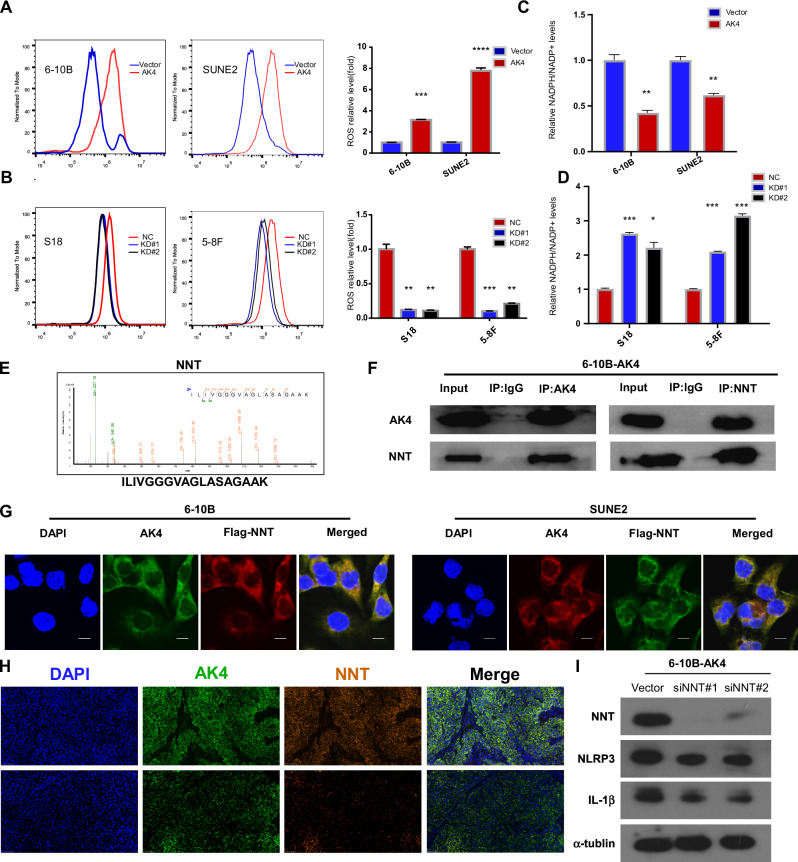
AK4 regulates NLRP3 signaling by binding to NNT. AK4 overexpression increased ROS levels and vice versa (**A**, **B**). AK4 overexpression suppressed intracellular NADPH/NADP+ levels and vice versa (**C**, **D**). Representative mass spectrometry plots and sequences of peptides from AK4 (**E**). Immunoprecipitation assay showing the interaction between AK4 and NNT (**F**). Immunofluorescence staining revealed that endogenous AK4 co-localized with NNT in the cytoplasm of NPC cells (**G**). Multiplex immunofluorescence staining revealed that AK4 co-localized with NNT in NPC biopsies (**I**). G scale bar: 10 μm.

Previous studies have reported that NLRP3 inflammasomes can be activated by diverse ligands and stimuli such as ATP, low intracellular K + , and high ROS [36–38]. Our results indicated that AK4 overexpression increased ROS levels and vice versa (Fig. 8A, B). A previous study demonstrated that the enhanced ROS response was mainly regulated by NADPH oxidase, and our results showed that intracellular NADPH/NADP+ levels were affected by AK4 overexpression or suppression in NPC cells (Fig. 8C, D). Moreover, NADPH generation is regulated by NNT, which catalyzes the transfer of hydride between NADH and NADP^+^ [39]. Interestingly, using IP and mass spectrometry, we found that the NNT protein strongly interacted with AK4 (Fig. 8E, F). Immunofluorescence staining revealed that endogenous AK4 co-localized with NNT in the cytoplasm of NPC cells (Fig. 8G, H). Additionally, NNT-targeting siRNAs were transfected into 6-10B cells stably overexpressing AK4; western blotting showed that NNT knockdown significantly downregulated NLRP3 and IL-1β (Fig. 8I). Based on the above results, we speculated that AK4 might bind to the NNT protein and regulate the generation of NADPH and, subsequently, ROS, which plays a vital role in NLRP3 inflammasome activation.

## Discussion

Distant metastasis and chemoresistance are major obstacles to improving the prognosis of patients with NPC [4]. In this study, we demonstrated that AK4 promoted NPC metastasis and chemoresistance by promoting IL-1β release by regulating the NLRP3 inflammatory complex.

Our data showed that AK4 significantly enhanced cell migration and promoted NPC metastasis, both in vitro and in vivo. Jan et al. demonstrated that AK4 promotes lung cancer metastasis by downregulating the expression of the transcription factor ATF3 and enhancing HIF-1α stability and EMT under hypoxia [12, 23]. However, we identified a novel pro-metastasis mechanism of AK4 in NPC—AK4 promoted NPC cell metastasis by regulating IL-1β secretion. Consistent with a previous study that AK4 could regulate the expression of inflammatory genes, including IL-1β, IL-6, and TNF-α, in M1 macrophages [40], our study also suggested that IL-1β was a downstream target affected by AK4 in NPC cells. We demonstrated that patients with NPC and a higher serum IL-1β concentration are prone to metastasis, and IL-1β can promote the migration of NPC cells in a concentration-dependent manner. Furthermore, the inhibition of IL-1β signaling using IL-1Ra partially abrogated AK4-promoted migration, invasion, and chemoresistance, indicating that IL-1β signaling is a functional mediator of the ability of AK4 to promote biological phenotypes both in vitro and in vivo. IL-1β is a prominent tumorigenic inflammatory marker that can trigger a cascade of inflammatory mediators, and extensive preclinical data are available to support the role of IL-1β in various stages of cancer development and progression, including tumor initiation, promotion, angiogenesis, and metastasis [41–43]. In addition, research has shown that chronic IL-1β-induced inflammation regulates EMT memory phenotypes *via* epigenetic modifications in non-small cell lung cancer [41–43]. In recent years, many basic and translational studies have investigated the development and use of novel IL-1β blockers in cancer therapy. IL-1β blockers, such as canakinumab and anakinra, have already been approved. They are used for the treatment of some autoimmune and autoinflammatory diseases and are currently being tested in preclinical and human clinical trials for cancer therapy [44, 45]. Together, our findings demonstrated that IL-1β inhibition might be an attractive therapeutic target in NPC, and further clinical trials are needed to confirm the efficacy and safety of IL-1β blockers in NPC. However, how AK4 regulates the expression of IL-1β is still unclear.

Previous studies have indicated that IL-1β was mainly produced by the NLRP3 inflammasome complex, which comprised NLRP3, together with molecules such as the adaptor protein ASC, which promote caspase-1 activation, thereby promoting IL-1β release [35]. Western blotting revealed that AK4 overexpression activated the NLRP3 inflammasome. Increasing evidence suggests that NLRP3 inflammasome activation depends on ROS generation. All known NLRP3 activators generate ROS; conversely, inhibitors of ROS block inflammasome activation [36]. Additionally, a previous study has shown that higher AK4 levels in MCF-7 cells elicit an increased ROS generation [18]. Consistent with this, we found that AK4 overexpression increased ROS levels in NPC cells. Therefore, increasing the AK4-ROS-NLRP3 axis is crucial for NLRP3 inflammasome activation and subsequent IL-1β release. Given that AK4 contributes to chemoresistance and metastasis by activating the NLRP3 inflammasome, AK4 may be a promising novel molecular target for NPC.

Our study found that AK4 strongly interacts with NNT, which is vital for the generation of NADPH and enhancement of ROS response. Additionally, knockdown of NNT significantly downregulated the expression of NLRP3 and IL-1β. Based on the above results, we speculated that AK4 might bind to the NNT protein and regulate the generation of NADPH and, subsequently ROS, which plays a vital role in NLRP3 inflammasome activation. However, the exact binding sites of AK4 and NNT were not determined. Regardless of the binding site, this study demonstrates that AK4 upregulation in NPC confers chemoresistance and metastasis by activating the NLRP3 pathway. AK4 holds promise as a novel molecular target and potential prognostic biomarker for NPC.

## Conclusion

In summary, this study showed that upregulation of AK4 conferred chemoresistance and promoted metastasis in NPC *via* IL-1β by regulating the NLRP3 inflammatory complex. Additionally, high IL-1β expression was associated with advanced clinical stage and was a prognostic factor associated with significantly poorer overall survival. These results may lead to novel therapeutic approaches for patients with NPC.

## Supplementary information

Supplement information

Western uncut

## Data Availability

All data generated or analyzed during this study are included in this published article and its supplementary files.

## References

[1] Bray F, Laversanne M, Sung H, Ferlay J, Siegel RL, Soerjomataram I, et al. Global cancer statistics 2022: GLOBOCAN estimates of incidence and mortality worldwide for 36 cancers in 185 countries. CA Cancer J Clin. 2024;74:229–63. 38572751 10.3322/caac.21834

[2] Wee JT, Ha TC, Loong SL, Qian CN. Is nasopharyngeal cancer really a “Cantonese cancer”? Chin J Cancer. 2010;29:517–26. 20426903 10.5732/cjc.009.10329

[3] Lai SZ, Li WF, Chen L, Luo W, Chen YY, Liu LZ, et al. How does intensity-modulated radiotherapy versus conventional two-dimensional radiotherapy influence the treatment results in nasopharyngeal carcinoma patients? Int J Radiat Oncol Biol Phys. 2011;80:661–8. 20643517 10.1016/j.ijrobp.2010.03.024

[4] Liu LT, Tang LQ, Chen QY, Zhang L, Guo SS, Guo L, et al. The prognostic value of plasma Epstein-Barr Viral DNA and tumor response to neoadjuvant chemotherapy in advanced-stage nasopharyngeal carcinoma. Int J Radiat Oncol Biol Phys. 2015;93:862–9. 26530755 10.1016/j.ijrobp.2015.08.003

[5] Tang XR, Li YQ, Liang SB, Jiang W, Liu F, Ge WX, et al. Development and validation of a gene expression-based signature to predict distant metastasis in locoregionally advanced nasopharyngeal carcinoma: a retrospective, multicentre, cohort study. Lancet Oncol. 2018;19:382–93. 29428165 10.1016/S1470-2045(18)30080-9

[6] Liu SL, Sun XS, Chen QY, Liu ZX, Bian LJ, Yuan L, et al. Development and validation of a transcriptomics-based gene signature to predict distant metastasis and guide induction chemotherapy in locoregionally advanced nasopharyngeal carcinoma. Eur J Cancer. 2022;163:26–34. 35032814 10.1016/j.ejca.2021.12.017

[7] Dzeja P, Terzic A. Adenylate kinase and AMP signaling networks: metabolic monitoring, signal communication and body energy sensing. Int J Mol Sci. 2009;10:1729–72. 19468337 10.3390/ijms10041729 PMC2680645

[8] Panayiotou C, Solaroli N, Karlsson A. The many isoforms of human adenylate kinases. Int J Biochem Cell Biol. 2014;49:75–83. 24495878 10.1016/j.biocel.2014.01.014

[9] Noma T, Fujisawa K, Yamashiro Y, Shinohara M, Nakazawa A, Gondo T, et al. Structure and expression of human mitochondrial adenylate kinase targeted to the mitochondrial matrix. Biochem J. 2001;358:225–32. 11485571 10.1042/0264-6021:3580225 PMC1222051

[10] Liu R, Ström AL, Zhai J, Gal J, Bao S, Gong W, et al. Enzymatically inactive adenylate kinase 4 interacts with mitochondrial ADP/ATP translocase. Int J Biochem Cell Biol. 2009;41:1371–80. 19130895 10.1016/j.biocel.2008.12.002 PMC2676352

[11] Liu R, Ström A, Zhai J, Gal J, Bao S, Gong W, et al. Enzymatically inactive adenylate kinase 4 interacts with mitochondrial ADP/ATP translocase. Int J Biochem cell Biol. 2009;41:1371–80. 19130895 10.1016/j.biocel.2008.12.002 PMC2676352

[12] Jan YH, Tsai HY, Yang CJ, Huang MS, Yang YF, Lai TC, et al. Adenylate kinase-4 is a marker of poor clinical outcomes that promotes metastasis of lung cancer by downregulating the transcription factor ATF3. Cancer Res. 2012;72:5119–29. 23002211 10.1158/0008-5472.CAN-12-1842

[13] Lanning NJ, Looyenga BD, Kauffman AL, Niemi NM, Sudderth J, DeBerardinis RJ, et al. A mitochondrial RNAi screen defines cellular bioenergetic determinants and identifies an adenylate kinase as a key regulator of ATP levels. Cell Rep. 2014;7:907–17. 24767988 10.1016/j.celrep.2014.03.065 PMC4046887

[14] Huang M, Qin X, Wang Y, Mao F. Identification of AK4 as a novel therapeutic target for serous ovarian cancer. Oncol Lett. 2020;20:346. 33123257 10.3892/ol.2020.12209 PMC7583734

[15] Jan YH, Lai TC, Yang CJ, Huang MS, Hsiao M. A co-expressed gene status of adenylate kinase 1/4 reveals prognostic gene signature associated with prognosis and sensitivity to EGFR targeted therapy in lung adenocarcinoma. Sci Rep. 2019;9:12329. 31444368 10.1038/s41598-019-48243-9 PMC6707279

[16] Fujisawa K, Terai S, Takami T, Yamamoto N, Yamasaki T, Matsumoto T, et al. Modulation of anti-cancer drug sensitivity through the regulation of mitochondrial activity by adenylate kinase 4. J Exp Clin Cancer Res. 2016;35:48. 26980435 10.1186/s13046-016-0322-2 PMC4793738

[17] Wu Z, Gong Q, Yu Y, Zhu J, Li W. Knockdown of circ-ABCB10 promotes sensitivity of lung cancer cells to cisplatin via miR-556-3p/AK4 axis. BMC Pulm Med. 2020;20:10. 31931771 10.1186/s12890-019-1035-z PMC6958770

[18] Liu X, Gonzalez G, Dai X, Miao W, Yuan J, Huang M, et al. Adenylate kinase 4 modulates the resistance of breast cancer cells to tamoxifen through an m(6)A-based epitranscriptomic mechanism. Mol Ther. 2020;28:2593–604. 32956623 10.1016/j.ymthe.2020.09.007 PMC7704734

[19] Lei W, Yan C, Ya J, Yong D, Yujun B, Kai L. MiR-199a-3p affects the multi-chemoresistance of osteosarcoma through targeting AK4. BMC Cancer. 2018;18:631. 29866054 10.1186/s12885-018-4460-0 PMC5987492

[20] Chen Y, Bao C, Zhang X, Lin X, Fu Y. Knockdown of LINC00662 represses AK4 and attenuates radioresistance of oral squamous cell carcinoma. Cancer Cell Int. 2020;20:244. 32549791 10.1186/s12935-020-01286-9 PMC7296632

[21] Xin F, Yao DW, Fan L, Liu JH, Liu XD. Adenylate kinase 4 promotes bladder cancer cell proliferation and invasion. Clin Exp Med. 2019;19:525–34. 31463832 10.1007/s10238-019-00576-5

[22] Zhang J, Yin YT, Wu CH, Qiu RL, Jiang WJ, Deng XG, et al. AK4 promotes the progression of HER2-positive breast cancer by facilitating cell proliferation and invasion. Dis Markers. 2019;2019:8186091. 31827645 10.1155/2019/8186091 PMC6886328

[23] Jan YH, Lai TC, Yang CJ, Lin YF, Huang MS, Hsiao M. Adenylate kinase 4 modulates oxidative stress and stabilizes HIF-1α to drive lung adenocarcinoma metastasis. J Hematol Oncol. 2019;12:12. 30696468 10.1186/s13045-019-0698-5 PMC6352453

[24] Liu L, Liu S, Deng P, Liang Y, Xiao R, Tang LQ, et al. Targeting the IRAK1-S100A9 Axis Overcomes Resistance to Paclitaxel in Nasopharyngeal Carcinoma. Cancer Res. 2021;81:1413–25. 33402387 10.1158/0008-5472.CAN-20-2125

[25] Subramanian A, Tamayo P, Mootha VK, Mukherjee S, Ebert BL, Gillette MA, et al. Gene set enrichment analysis: a knowledge-based approach for interpreting genome-wide expression profiles. Proc Natl Acad Sci USA. 2005;102:15545–50. 16199517 10.1073/pnas.0506580102 PMC1239896

[26] Cao J, Chen X, Jiang L, Lu B, Yuan M, Zhu D, et al. DJ-1 suppresses ferroptosis through preserving the activity of S-adenosyl homocysteine hydrolase. Nat Commun. 2020;11:1251. 32144268 10.1038/s41467-020-15109-y PMC7060199

[27] Dinarello CA. Interleukin-1 beta, interleukin-18, and the interleukin-1 beta converting enzyme. Ann NY Acad Sci. 1998;856:1–11. 9917859 10.1111/j.1749-6632.1998.tb08307.x

[28] Tulotta C, Lefley DV, Freeman K, Gregory WM, Hanby AM, Heath PR, et al. Endogenous production of IL1B by breast cancer cells drives metastasis and colonization of the bone microenvironment. Clin Cancer Res. 2019;25:2769–82. 30670488 10.1158/1078-0432.CCR-18-2202

[29] Wu TC, Xu K, Martinek J, Young RR, Banchereau R, George J, et al. IL1 receptor antagonist controls transcriptional signature of inflammation in patients with metastatic breast cancer. Cancer Res. 2018;78:5243–58. 30012670 10.1158/0008-5472.CAN-18-0413 PMC6391892

[30] Voronov E, Shouval DS, Krelin Y, Cagnano E, Benharroch D, Iwakura Y, et al. IL-1 is required for tumor invasiveness and angiogenesis. Proc Natl Acad Sci USA. 2003;100:2645–50. 12598651 10.1073/pnas.0437939100 PMC151394

[31] Yano S, Nokihara H, Yamamoto A, Goto H, Ogawa H, Kanematsu T, et al. Multifunctional interleukin-1beta promotes metastasis of human lung cancer cells in SCID mice via enhanced expression of adhesion-, invasion- and angiogenesis-related molecules. Cancer Sci. 2003;94:244–52. 12824917 10.1111/j.1349-7006.2003.tb01428.x PMC11160152

[32] Apte RN, Krelin Y, Song X, Dotan S, Recih E, Elkabets M, et al. Effects of micro-environment- and malignant cell-derived interleukin-1 in carcinogenesis, tumour invasiveness and tumour-host interactions. Eur J Cancer. 2006;42:751–9. 16530403 10.1016/j.ejca.2006.01.010

[33] Song X, Voronov E, Dvorkin T, Fima E, Cagnano E, Benharroch D, et al. Differential effects of IL-1 alpha and IL-1 beta on tumorigenicity patterns and invasiveness. J Immunol. 2003;171:6448–56. 14662844 10.4049/jimmunol.171.12.6448

[34] Yoshida N, Ikemoto S, Narita K, Sugimura K, Wada S, Yasumoto R, et al. Interleukin-6, tumour necrosis factor alpha and interleukin-1beta in patients with renal cell carcinoma. Br J Cancer. 2002;86:1396–400. 11986770 10.1038/sj.bjc.6600257 PMC2375361

[35] Agostini L, Martinon F, Burns K, McDermott MF, Hawkins PN, Tschopp J. NALP3 forms an IL-1beta-processing inflammasome with increased activity in Muckle-Wells autoinflammatory disorder. Immunity. 2004;20:319–25. 15030775 10.1016/s1074-7613(04)00046-9

[36] Dostert C, Pétrilli V, Van Bruggen R, Steele C, Mossman BT, Tschopp J. Innate immune activation through Nalp3 inflammasome sensing of asbestos and silica. Science. 2008;320:674–7. 18403674 10.1126/science.1156995 PMC2396588

[37] Pétrilli V, Papin S, Dostert C, Mayor A, Martinon F, Tschopp J. Activation of the NALP3 inflammasome is triggered by low intracellular potassium concentration. Cell Death Differ. 2007;14:1583–9. 17599094 10.1038/sj.cdd.4402195

[38] Mariathasan S, Newton K, Monack DM, Vucic D, French DM, Lee WP, et al. Differential activation of the inflammasome by caspase-1 adaptors ASC and Ipaf. Nature. 2004;430:213–8. 15190255 10.1038/nature02664

[39] Kampjut D, Sazanov LA. Structure and mechanism of mitochondrial proton-translocating transhydrogenase. Nature. 2019;573:291–5. 31462775 10.1038/s41586-019-1519-2

[40] Chin WY, He CY, Chow TW, Yu QY, Lai LC, Miaw SC. Adenylate kinase 4 promotes inflammatory gene expression via Hif1α and AMPK in macrophages. Front Immunol. 2021;12:630318. 33790902 10.3389/fimmu.2021.630318 PMC8005550

[41] Mantovani A, Dinarello CA, Molgora M, Garlanda C. Interleukin-1 and related cytokines in the regulation of inflammation and immunity. Immunity. 2019;50:778–95. 30995499 10.1016/j.immuni.2019.03.012 PMC7174020

[42] Apte RN, Voronov E. Immunotherapeutic approaches of IL-1 neutralization in the tumor microenvironment. J Leukoc Biol. 2017;102:293–306. 28522598 10.1189/jlb.3MR1216-523R

[43] Grivennikov SI, Greten FR, Karin M. Immunity, inflammation, and cancer. Cell. 2010;140:883–99. 20303878 10.1016/j.cell.2010.01.025 PMC2866629

[44] Wong CC, Baum J, Silvestro A, Beste MT, Bharani-Dharan B, Xu S, et al. Inhibition of IL1β by canakinumab may be effective against diverse molecular subtypes of lung cancer: an exploratory analysis of the CANTOS trial. Cancer Res. 2020;80:5597–605. 33023946 10.1158/0008-5472.CAN-19-3176

[45] Kopp WC, Urba WJ, Rager HC, Alvord WG, Oppenheim JJ, Smith JW, et al. Induction of interleukin 1 receptor antagonist after interleukin 1 therapy in patients with cancer. Clin Cancer Res. 1996;2:501–6. 9816196

