# Supplementary material for: AK4 promotes nasopharyngeal carcinoma metastasis and chemoresistance by activating NLRP3 inflammatory complex
Source: Cell Death Dis. 2025 Jul 1;16(1):480. doi: 10.1038/s41419-025-07805-8 (PMC12217281; doi:10.1038/s41419-025-07805-8)

Supplementary table 1. Primers and oligonucleotides

| **Used for RT-PCR** | |
| --- | --- |
| GAPDH-up | CTGGGCTACACTGAGCACC |
| GAPDH-dn | AAGTGGTCGTTGAGGGCAATG |
| AK4-up | TCACACGCCTAATGATGTCCG |
| AK4-dn | CGGCTGAGACGATCTTTAAGTG |
| IL-1β-up | ATGATGGCTTATTACAGTGGCAA |
| IL-1β-dn | GTCGGAGATTCGTAGCTGGA |
| **siRNA** | |
| siNLRP3#1 | GGATCAAACTACTCTGTGA |
| siNLRP3#2 | GAGAGACCTTTATGAGAAA |
| siNNT#1 | TCGTTATCACTGTGCTGAA |
| siNNT#2 | CTATGGTTAATCCAACATT |
| **AK4** |  |
| EX-NEG-Lv201-up | GCGGTAGGCGTGTACGGT |
| EX-NEG-Lv201-dn | CTGGAATAGCTCAGAGGC |
| pEZ-Lv201-puro-AK4-up | TGGAAGGAGTTCGAACCATGGCTTCCAAACTCC |
| pEZ-Lv201-puro-AK4-dn | GCGGCCGCACTCGAGCTAATATGCTTCTTTGGAC |
| **ShRNA** |  |
| psi-LVRU6GP-up | TAATACGACTCACTATAGGG |
| psi-LVRU6GP-dn | CTGGAATAGCTCAGAGGC |
| psi-LVRU6GP-AK4#1 | gatccgGGATCTAGTGATCAGTTTGAATCAAGAGTTCAAACTGATCACTAGATCCttttttg |
| psi-LVRU6GP-AK4#2 | gatccgGGGTATATAACCTGGACTTCATCAAGAGTGAAGTCCAGGTTATATACCCttttttg |

Supplementary table 2: Association between AK4 expression and the clinicopathological features of 191 patients with NPC.

|  | Patients (n) | AK4 | | *P* |
| --- | --- | --- | --- | --- |
|  |  | Low level | High level |  |
| Age(years) |  |  |  | 0.569 |
| <45 | 98 (51.3) | 87 (52.4) | 11 (44.0) |  |
| ≥45 | 93 (48.7) | 79 (47.6) | 14 (56.0) |  |
| Gender |  |  |  | 0.812 |
| Female | 61 (31.9) | 52 (31.3) | 9 (36.0) |  |
| Male | 130 (68.1) | 114 (68.7) | 16 (64.0) |  |
| T stage* |  |  |  | 0.336 |
| T1-2 | 43 (22.5) | 35 (21.1) | 8 (32.0) |  |
| T3-4 | 148 (77.5) | 131 (78.9) | 17 (68.0) |  |
| N stage* |  |  |  | 0.632 |
| N0-1 | 87 (45.5) | 74 (44.6) | 13 (52.0) |  |
| N2-3 | 104 (54.5) | 92 (55.4) | 12 (48.0) |  |
| TNM* |  |  |  | 1.000 |
| II-III | 125 (65.4) | 109 (65.7) | 16 (64.0) |  |
| IV | 66 (34.6) | 57 (34.3) | 9 (36.0) |  |
| EBV DNA |  |  |  | 1.000 |
| ≥1500 | 102 (53.4) | 89 (53.6) | 13 (52.0) |  |
| <1500 | 89 (46.6) | 77 (46.4) | 12 (48.0) |  |

EBV, Epstein–Barr virus.

*According to the 8th edition of UICC/AJCC staging system.

Supplementary table 3: Association between IL-1β expression and the clinicopathological features of 262 patients with NPC.

|  | Patients (n) | IL-1β | | *P* |  |
| --- | --- | --- | --- | --- | --- |
|  |  | Low level | High level |  |  |
| Age(years) |  |  |  | 0.844 |  |
| < 45 | 142 (54.2) | 61 (43.0) | 81 (57.0) |  |  |
| ≥ 45 | 120 (45.8) | 53 (44.2) | 67 (55.8) |  |  |
| Sex |  |  |  | 0.502 |  |
| Male | 204 (77.9) | 91(44.6) | 113 (55.4) |  |  |
| Female | 58 (22.1) | 23 (39.7) | 35 (60.3) |  |  |
| Pathology type |  |  |  | 0.254 |  |
| WHO II | 1 (0.4) | 1 (100.0) | 0 (0.0) |  |  |
| WHO III | 261 (99.6) | 113 (43.3) | 148 (56.7) |  |  |
| T stage* |  |  |  | 0.035 |  |
| T1-2 | 35 (13.4) | 21 (60.0) | 14 (40.0) |  |  |
| T3-4 | 227 (86.6) | 93 (41.0) | 134 (59.0) |  |  |
| N stage* |  |  |  | 0.699 |  |
| N0-1 | 84 (32.1) | 38 (45.2) | 46 (54.8) |  |  |
| N2-3 | 178 (67.9) | 76 (42.7) | 102 (57.3) |  |  |
| TNM stage* |  |  |  | 0.503 |  |
| II-III | 102 (38.9) | 47 (46.1) | 55 (53.9) |  |  |
| IV | 160 (61.1) | 67 (41.9) | 93 (58.1) |  |  |
| EBV DNA |  |  |  | 0.206 |  |
| ≥ 1500 | 163 (62.2) | 66 (40.5) | 97 (59.5) |  |  |
| < 1500 | 99 (37.8) | 48 (48.5) | 51 (51.5) |  |  |

WHO, World Health Organization; EBV, Epstein–Barr virus.

*According to the 8th edition of UICC/AJCC staging system.

Supplementary table 4: Multivariate Cox regression analysis of the associations between various clinicopathological features and survival in 262 patients with NPC.

|  | Hazard ratio*  (95% CI) | P  value |
| --- | --- | --- |
| Distant metastasis-free survival |  |  |
| Age (y) (≥ 45 vs. < 45) | 0.643(0.286-1.443) | 0.284 |
| Gender (M vs. F) | 1.978 (0.667-5.865) | 0.219 |
| T category (3-4 vs. 1-2) | 0.419 (0.163-1.080) | 0.072 |
| N category (2-3 vs. 0-1) | 1.154 (0.464-2.873) | 0.757 |
| Overall stage (IV vs. II-III) | 0.972 (0.424-2.229) | 0.946 |
| EBV DNA (≥1500 vs. <1500) | 1.657 (0.670-4.099) | 0.275 |
| IL-1β (high vs. low) | 5.768 (1.959-16.984) | 0.001 |
| Progression-free survival |  |  |
| Age (y) (≥ 45 vs. < 45) | 0.822 (0.445-1.519) | 0.531 |
| Gender (M vs. F) | 2.068 (0.860-4.971) | 0.105 |
| T category (3-4 vs. 1-2) | 0.584 (0.265-1.290) | 0.184 |
| N category (2-3 vs. 0-1) | 0.800 (0.404-1.584) | 0.523 |
| Overall stage (IV vs. II-III) | 1.555 (0.785-3.079) | 0.206 |
| EBV DNA (≥1500 vs. <1500) | 1.614 (0.792-3.291) | 0.188 |
| IL-1β (high vs. low) | 2.313 (1.199-4.463) | 0.012 |
| Overall survival |  |  |
| Age (y) (≥ 45 vs. < 45) | 1.935 (0.719-5.205) | 0.191 |
| Gender (M vs. F) | 0.790 (0.244-2.552) | 0.693 |
| T category (3-4 vs. 1-2) | 1.110 (0.243-5.081) | 0.893 |
| N category (2-3 vs. 0-1) | 1.730 (0.522-5.734) | 0.370 |
| Overall stage (IV vs. II-III) | 2.082 (0.656-6.613) | 0.213 |
| EBV DNA (≥1500 vs. <1500) | 0.913 (0.304-2.738) | 0.871 |
| IL-1β (high vs. low) | 3.637 (1.143-11.577) | 0.029 |

A Cox proportional hazards regression model was used to detect variables individually without adjustment. All variables were transformed into categorical variables. HRs were calculated for age (years) (≥45 vs. <45), sex (male vs. female), T stage (T3-4 vs. T1-2), N stage (N2-3 vs. N0-1), overall stage (IVa-b vs. II-III), plasma EBV DNA before the first treatment (≥1500 copies/ml vs. <1500 copies/ml), and plasma IL-1β (>31.3 pg/ml vs. ≤31.3 pg/ml).

**Supplementary figure 1:** The expression of three other genes in array express microarray data and The Cancer Genome Atlas (head and neck) microarray data. Expression of CPAMD8, DDAH1, and CRTR1 in Array express microarray data (GEO accession number: [GSE12452](https://www.ncbi.nlm.nih.gov/protein/GSE12452), GSE53819; http://www.ncbi.nlm.nih.gov/geo/) (A, B) and The Cancer Genome Atlas (head and neck) tumor and normal tissue microarray data (C). Kaplan–Meier overall survival curves for patients from The Cancer Genome Atlas (head and neck) stratified by high versus low AK4 expression (http://www.oncolnc.org/kaplan/?lower=50&upper=50&cancer=HNSC&gene_id=205&raw=AK4&species=mRNA) (D). Kaplan-Meier distant metastasis free survival for patients with NPC stratiﬁed by high versus low AK4 expression (E). P-values were calculated using the log-rank test. Student’s t-test. * P < 0.05; ** P < 0.01; *** P < 0.001; **** P < 0.0001.


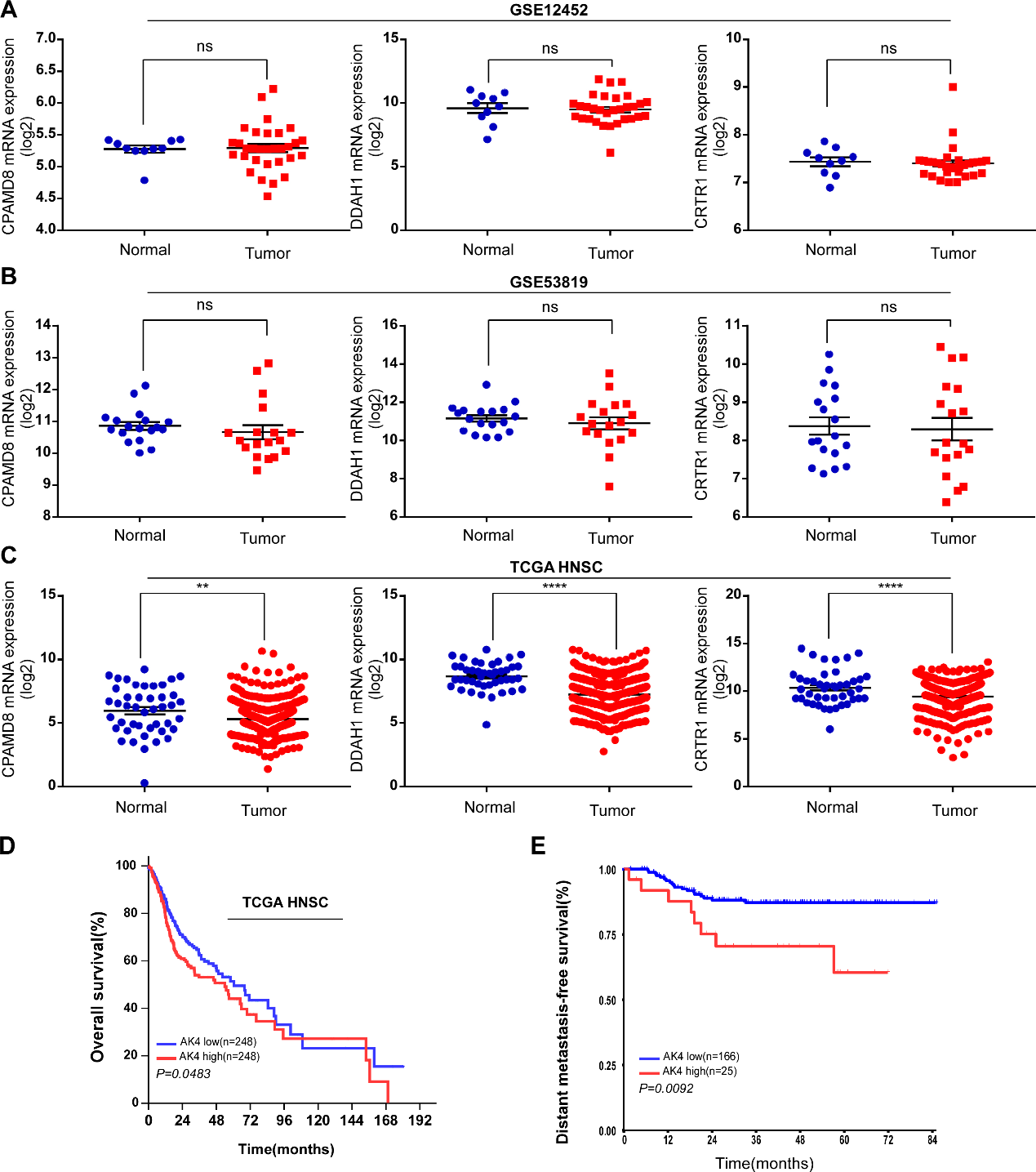


**Supplementary figure 2:** AK4 expression correlates with IL-1β in NPC. Microarray analysis (GSE13597, GSE53819, and TCGA) revealed that AK4 expression was positively correlated with IL-1β (A, B, C). (A) IL-1β expression was positively correlated to AK4 expression in 47 primary human NPC specimens. Two representative cases are illustrated (D, E). Western blotting and correlation analysis of AK4 and IL-1β expression levels in nine freshly-collected human NPC samples. α-tubulin was used as the loading control (F, G).


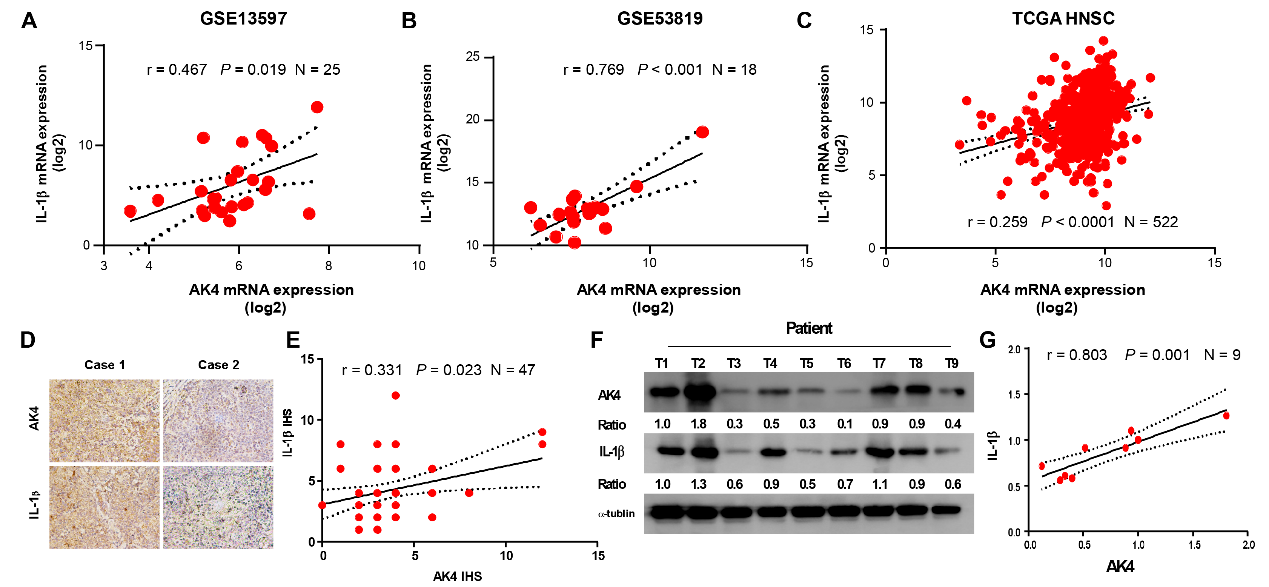

Supplement: Supplementary file 1 — Supplement information [file 41419_2025_7805_MOESM1_ESM.docx]
