# Supplementary material for: AK4 promotes nasopharyngeal carcinoma metastasis and chemoresistance by activating NLRP3 inflammatory complex
Source: Cell Death Dis. 2025 Jul 1;16(1):480. doi: 10.1038/s41419-025-07805-8 (PMC12217281; doi:10.1038/s41419-025-07805-8)

Figure 1

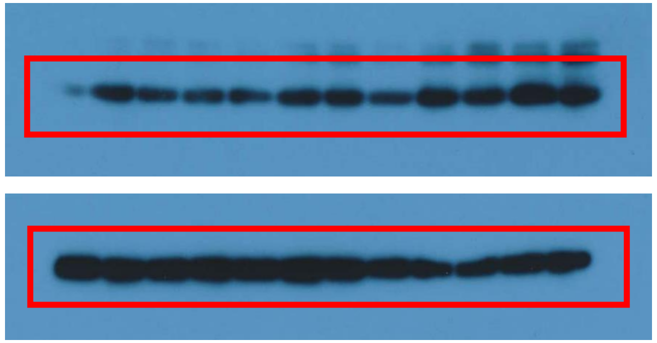

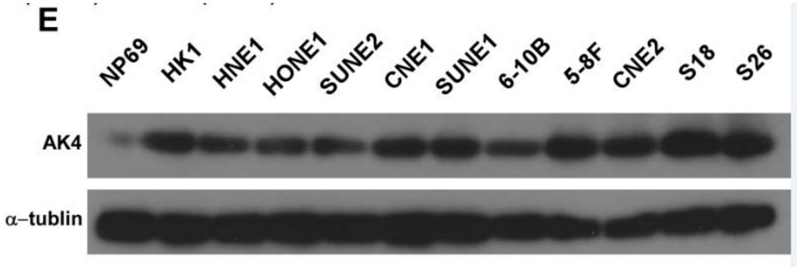
E
NP69
HK1
HNE1
HONE1
SUNE2
CNE1
SUNE1
6-10B
5-8F
CNE2
S18
S26
AK4
α–tublin

Figure 2

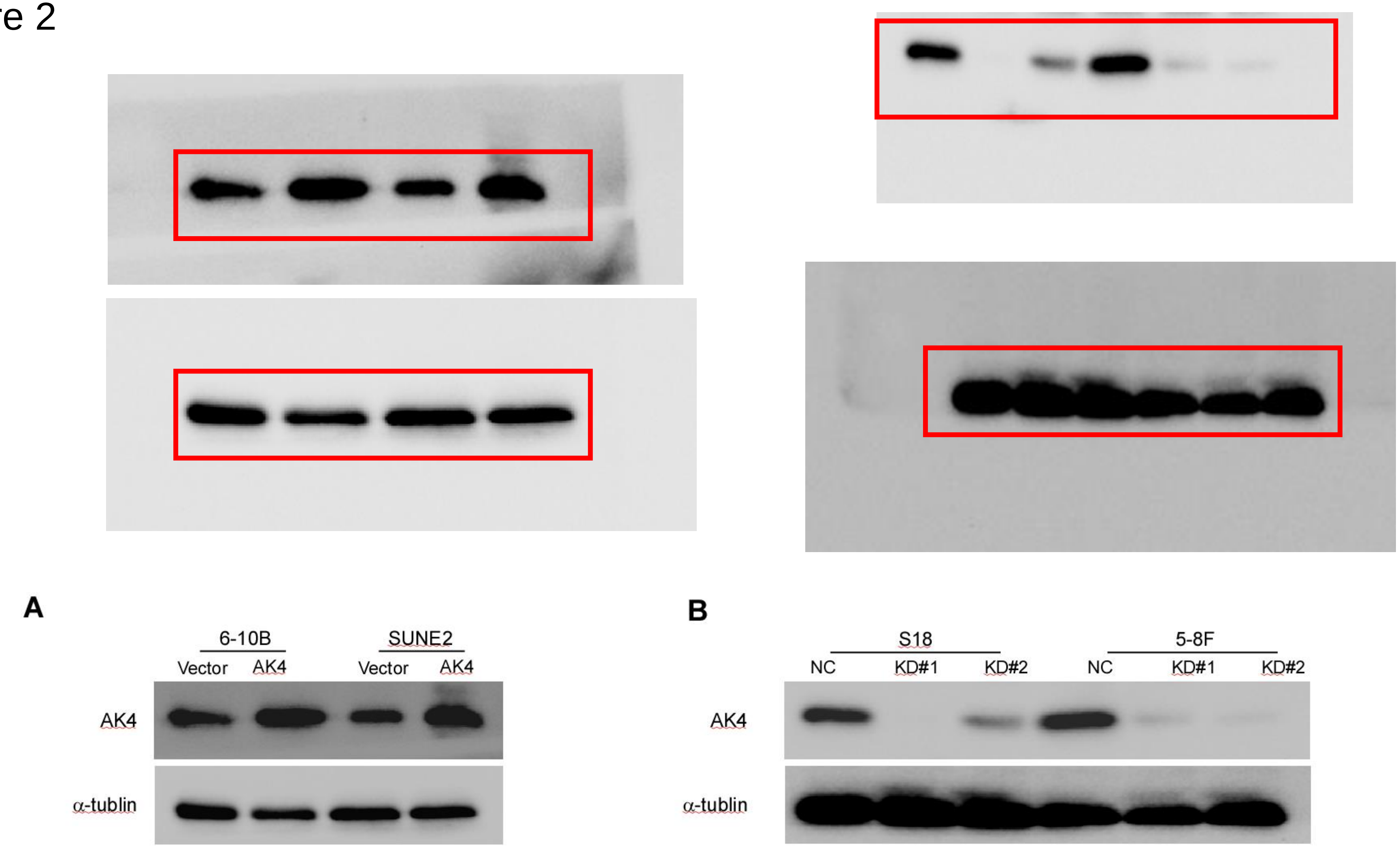
A
6-10B
SUNE2
Vector
AK4
Vector
AK4
AK4
α-tublin
B
S18
5-8F
NC
KD#1
KD#2
NC
KD#1
KD#2
AK4
α-tublin

Figure 3

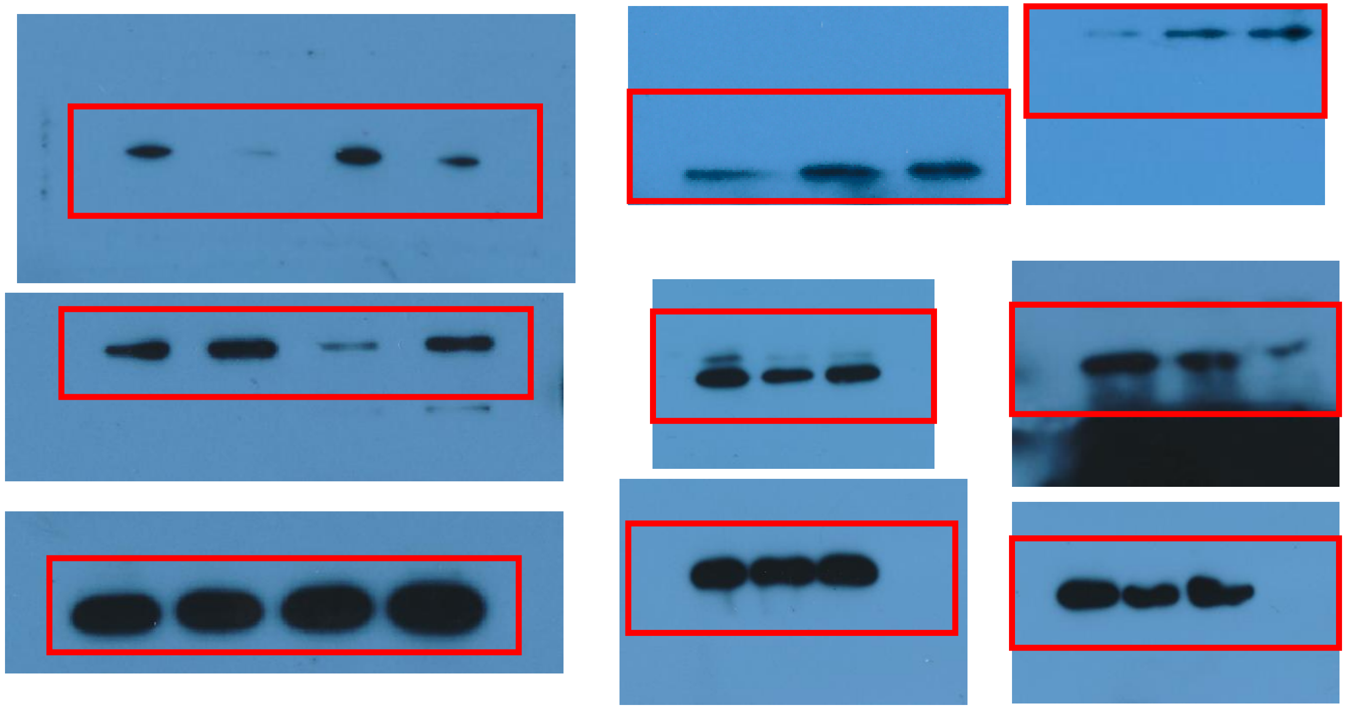

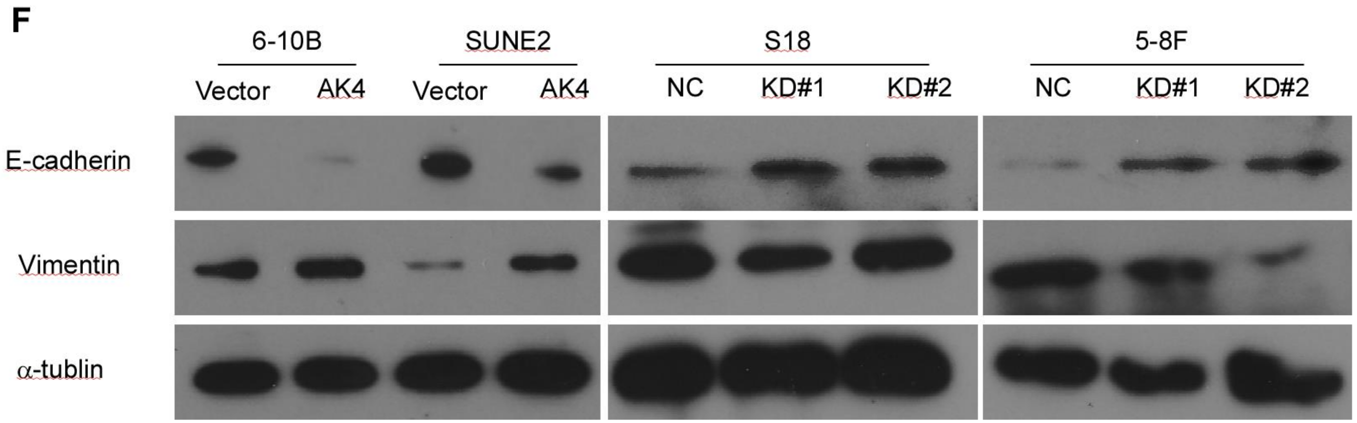
F
6-10B
SUNE2
S18
5-8F
Vector
AK4
Vector
AK4
NC
KD#1
KD#2
NC
KD#1
KD#2
E-cadherin
Vimentin
α-tublin

Figure 7

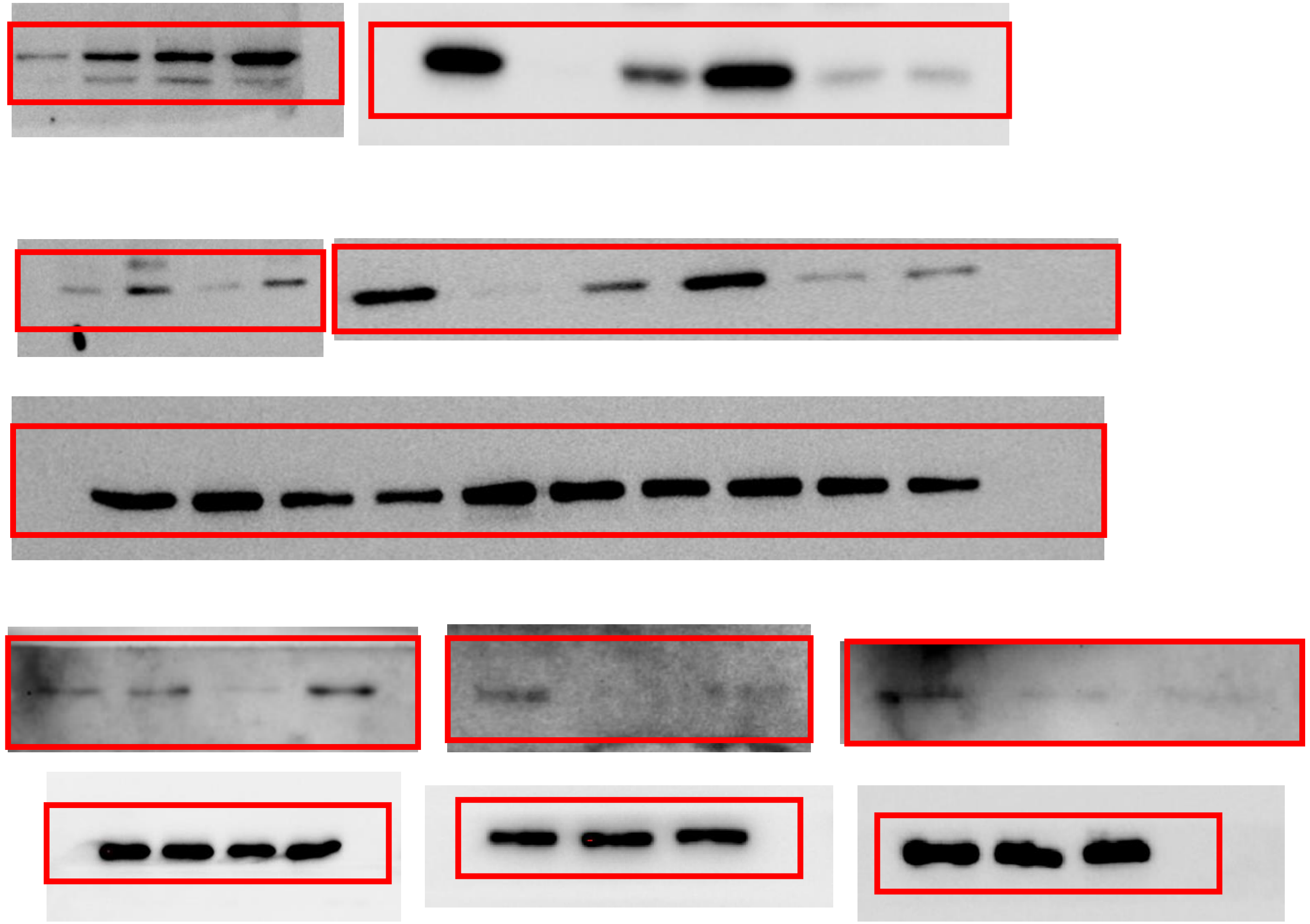

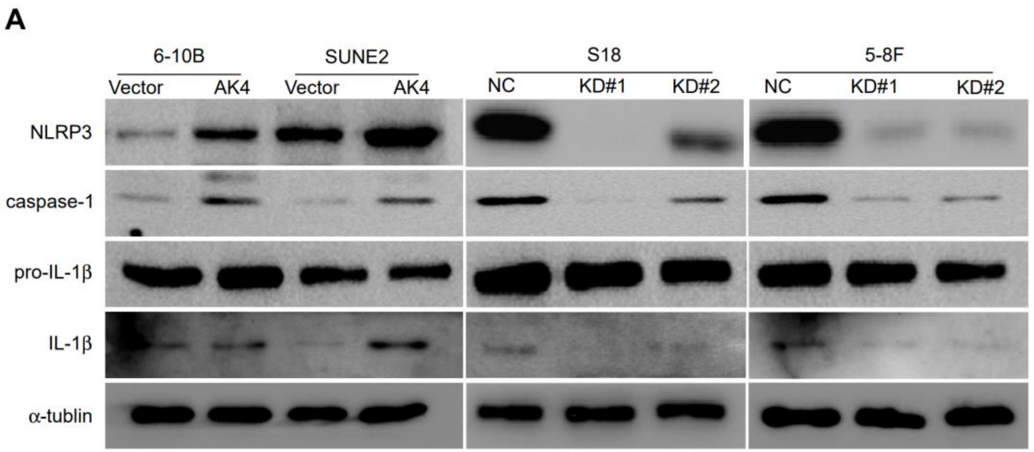
A
6-10B
SUNE2
S18
5-8F
Vector
AK4
Vector
AK4
NC
KD#1
KD#2
NC
KD#1
KD#2
NLRP3
caspase-1
pro-IL-1β
IL-1β
α-tublin

Figure 7

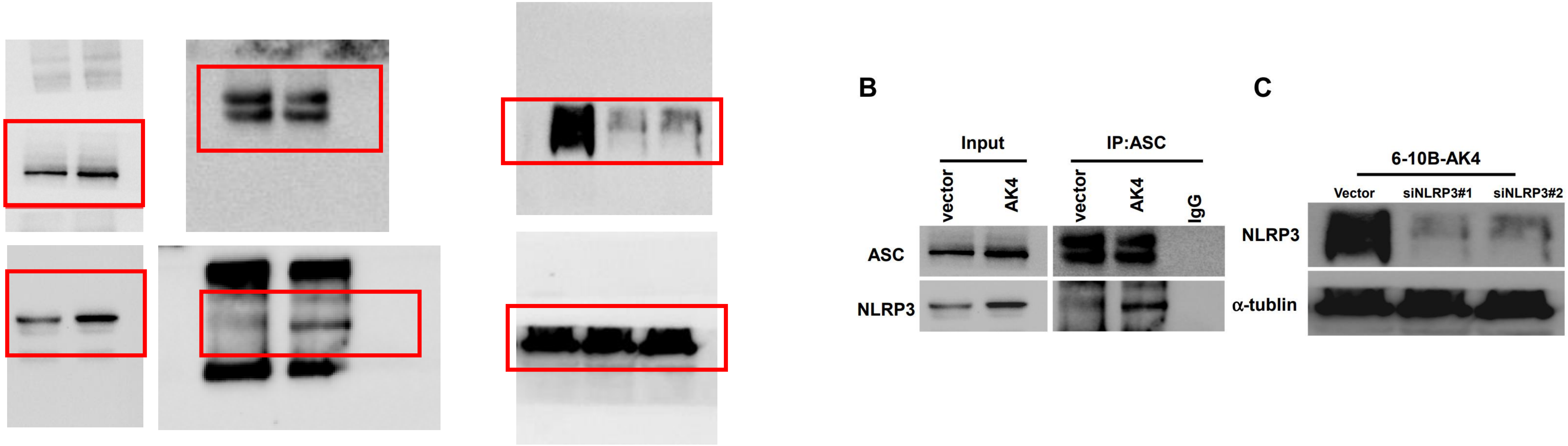
B
Input
IP:ASC
vector
AK4
vector
AK4
IgG
ASC
NLRP3
C
6-10B-AK4
Vector
siNLRP3#1
siNLRP3#2
NLRP3
α-tublin

Figure 8

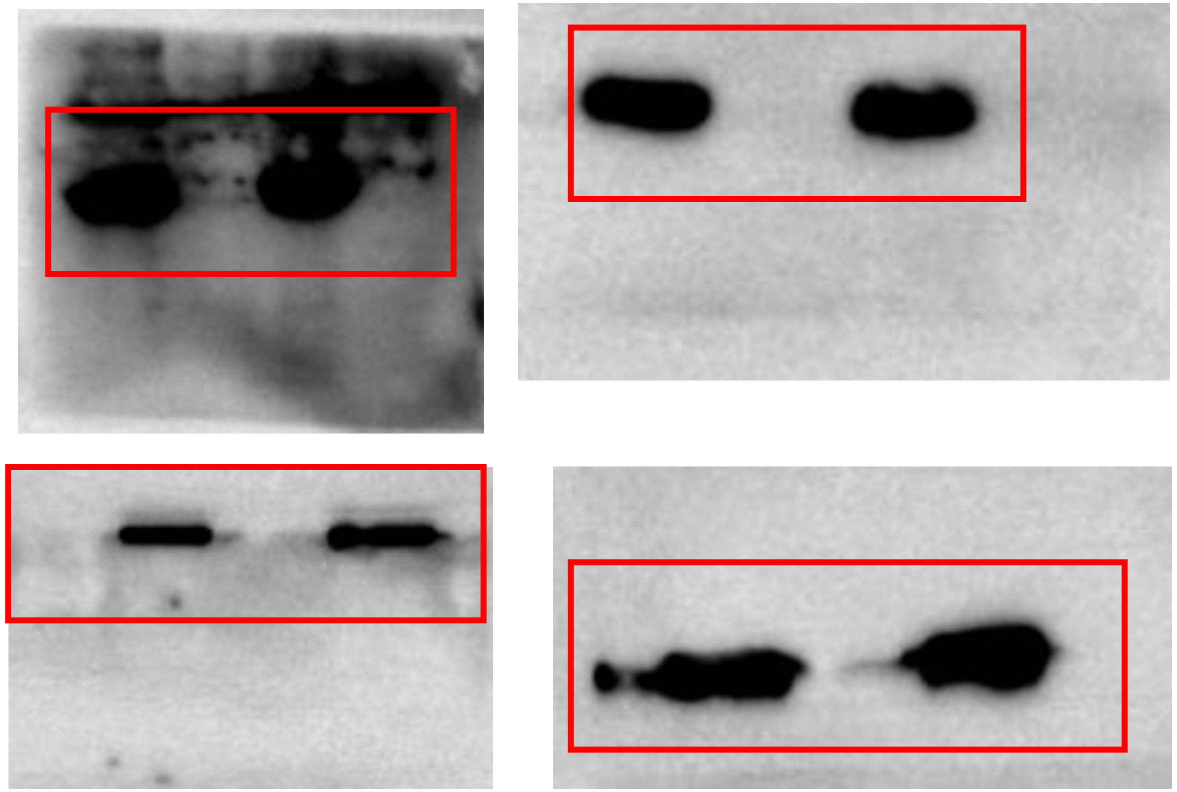

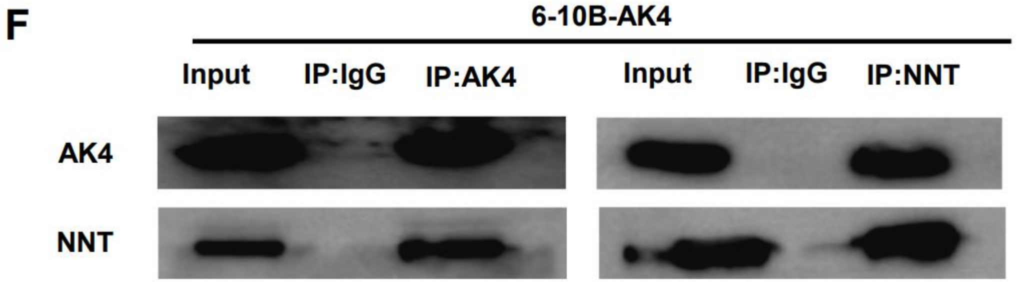

Figure 8

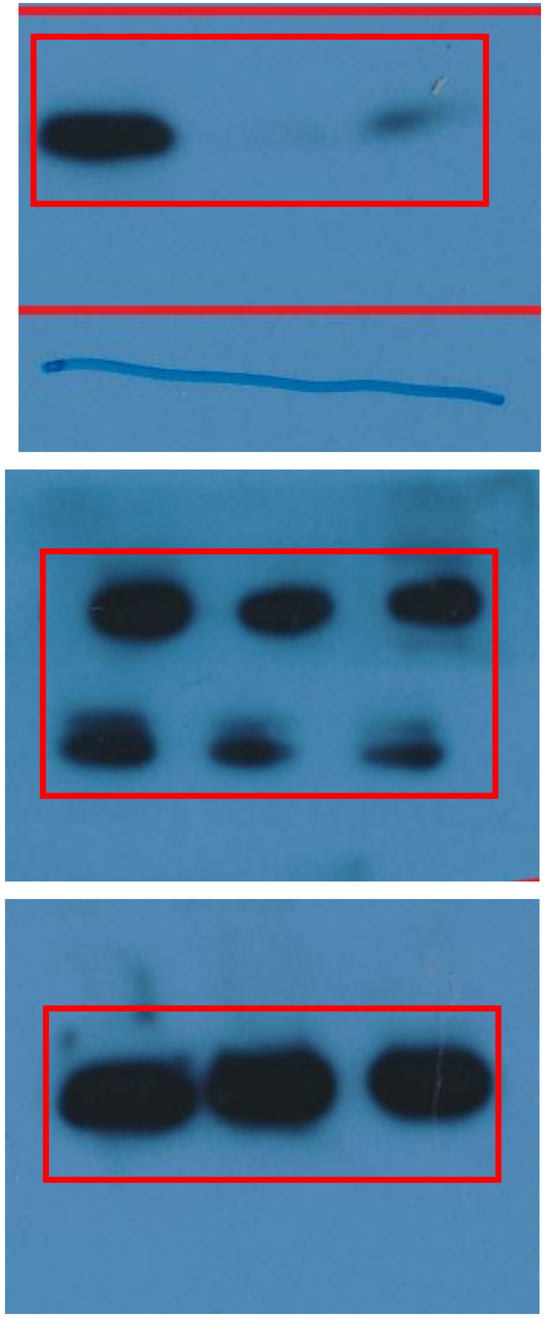

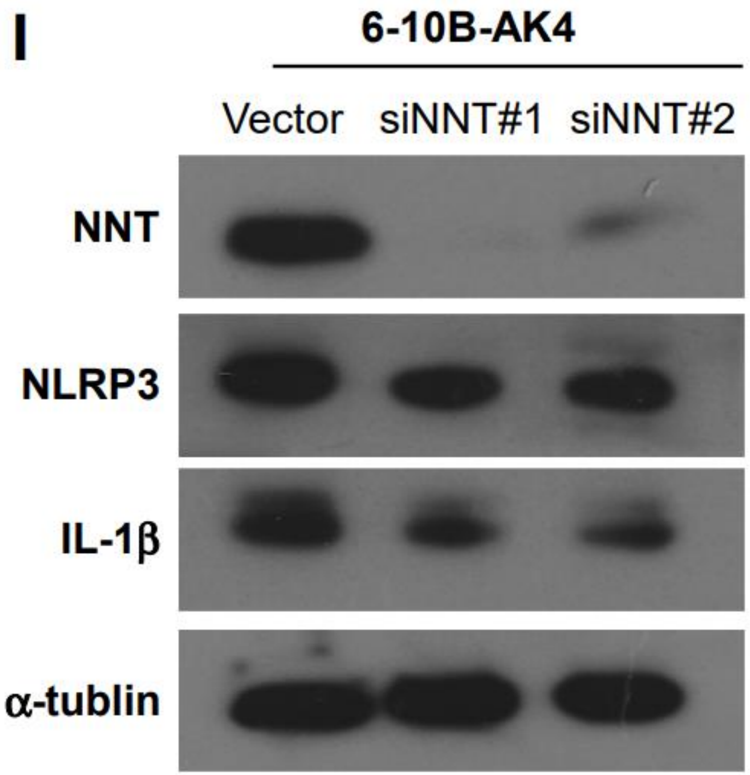

Supplement Figure 2

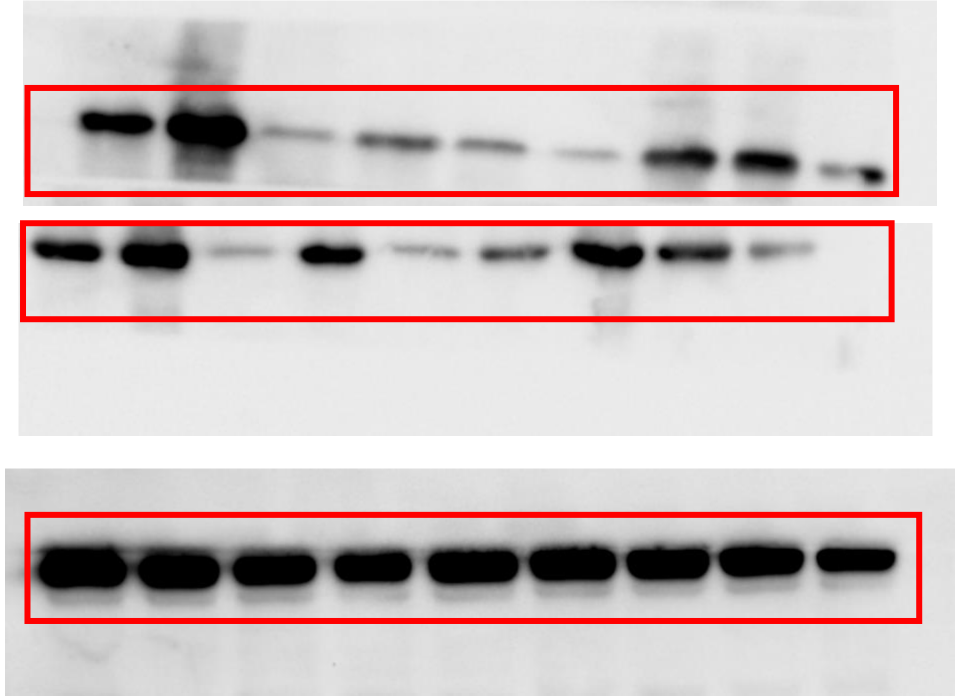

F

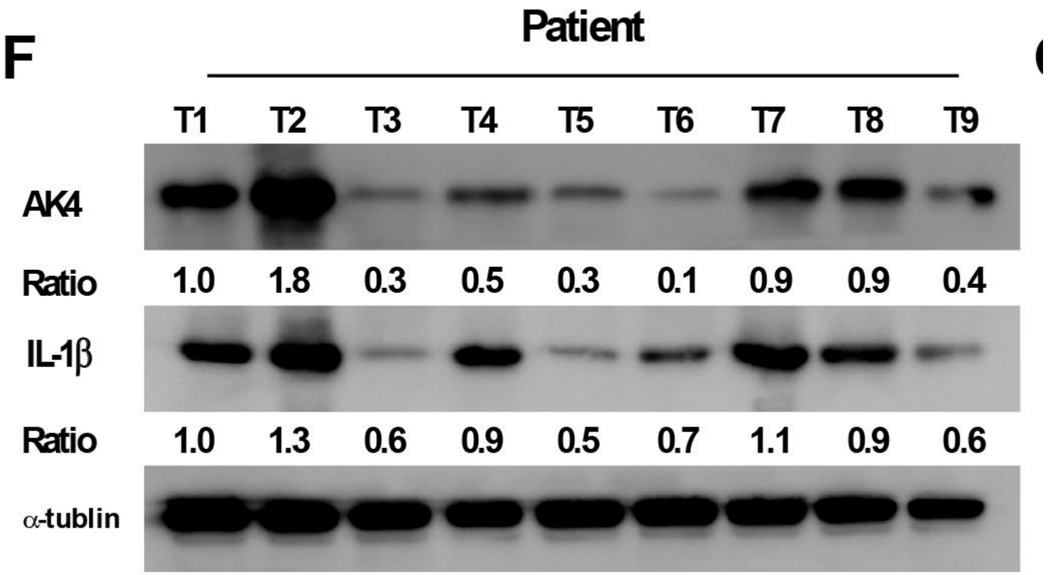

Supplement: Supplementary file 2 — Western uncut [file 41419_2025_7805_MOESM2_ESM.pdf]
